# Additive and mostly adaptive plastic responses of gene expression to multiple stress in *Tribolium castaneum*

**DOI:** 10.1371/journal.pgen.1008768

**Published:** 2020-05-07

**Authors:** Eva L. Koch, Frédéric Guillaume

**Affiliations:** 1 Department of Evolutionary Biology and Environmental Studies, University of Zürich, Zürich, Switzerland; 2 Department of Animal and Plant Science, University of Sheffield, Western Bank, Sheffield, United Kingdom; University of Georgia, UNITED STATES

## Abstract

Gene expression is known to be highly responsive to the environment and important for adjustment of metabolism but there is also growing evidence that differences in gene regulation contribute to species divergence and differences among locally adapted populations. However, most studies so far investigated populations when divergence had already occurred. Selection acting on expression levels at the onset of adaptation to an environmental change has not been characterized. Understanding the mechanisms is further complicated by the fact that environmental change is often multivariate, meaning that organisms are exposed to multiple stressors simultaneously with potentially interactive effects. Here we use a novel approach by combining fitness and whole-transcriptome data in a large-scale experiment to investigate responses to drought, heat and their combination in *Tribolium castaneum*. We found that fitness was reduced by both stressors and their combined effect was almost additive. Expression data showed that stressor responses were acting independently and did not interfere physiologically. Since we measured expression and fitness within the same individuals, we were able to estimate selection on gene expression levels. We found that variation in fitness can be attributed to gene expression variation and that selection pressures were environment dependent and opposite between control and stress conditions. We could further show that plastic responses of expression were largely adaptive, i.e. in the direction that should increase fitness.

## Author summary

During environmental change organisms are exposed to several stressors simultaneously to which they can show plastic and evolutionary responses. Gene regulation can play a crucial role in both processes. Measuring gene expression gives us detailed insights into underlying molecular pathways, relative importance of different stressors and how they may interfere with each other in combination. However, it is challenging to understand how plastic responses are ultimately linked to fitness and adaptation. We combined measurements of a fitness component (offspring number) with gene expression data to study responses of the model organism *T*. *castaneum* (red flour beetle) to single and joint effects of drought and heat. Higher temperatures resulted in a stronger reduction of offspring number and a larger number of differently expressed genes compared to reduced humidity. We could show that stressors affected different physiological processes and consequently responses did not show much modification in combination. Combining fitness and transcriptomic data of the same individuals enabled us to estimate selection on gene expression levels. We found that selection under control conditions (to which the beetles are adapted) was in general opposite to selection under the new, stressful conditions and that large parts of the response to heat were adaptive.

## Introduction

One of the major goals of evolutionary biology is to understand the genetic basis of phenotypic variation and how it is shaped by natural selection. The mapping of genetic to phenotypic variation depends on many cellular processes, of which mRNA abundance, or gene expression, has been shown to play a central role [1–4]. For variation in expression to be relevant for evolution it needs a heritable genetic basis and a link with fitness variation. While the heritability of expression variation has been established in many cases [5–10], we still lack direct estimates of the strength of selection acting on transcript level abundance. The link between expression levels and fitness variation is not obvious, since mRNA abundance must be translated into protein abundance, enzyme activity and ultimately phenotypic variation [11,12]. So far, the evidence for a link between fitness and gene expression variation is mixed. For instance, in yeast, the knocking-out of many genes had inconsequential effects on fitness [13], whereas more recent evidence showed that variation in expression can significantly affect fitness [14,15]. Unfortunately, data in more complex organisms are still scarce, especially on a transcriptome-wide scale. Indirect evidence supporting the importance of gene expression in evolution comes from studies showing differences in expression levels between adaptively divergent populations in yeast [16], humans [17], *Drosophila* [18], or fish [9,19–21]. In such cases, further support can be brought when evolved differences in regulatory DNA sequences are found [15,22]. Additionally, experimental evolution approaches in multiple organisms were successful to detect altered expression levels within a few generations that adapted to different environmental conditions [20,23–26].

Phenotypic plasticity can also play an important role in population differentiation, especially at the onset of adaptation to novel environments [27–29]. Studying the extent of plasticity in gene expression is particularly relevant because it is a highly plastic trait, often involved in the immediate response of organisms to changes in their environment [30–33]. The role of plasticity in evolution is, however, contentious. It is often argued that if plasticity is adaptive, it should impede evolution since it can hide genetic variance on which selection would act and thus weakens selection [34]. Yet, plasticity is also crucial for population persistence in a changing environment because it can keep populations at higher sizes, or buffer novel variants against purifying selection [28,35]. It may thus facilitate long-term adaptation [27,29,36] by maintaining higher genetic variance. It can also promote population divergence by allowing colonization of new habitats and exploitation of new niches [37,38].

To better understand the role of plasticity in the evolution of adaptive divergence in gene expression, we need to understand the short- and long-term fitness effects of plastic changes in mRNA abundance. Studies comparing plastic and evolved responses of gene expression in natural populations repeatedly found that plasticity was in opposite direction to the evolutionary response [19,20,39] and concluded that plastic changes were maladaptive. It may thus be that maladaptive plasticity facilitates evolutionary divergence by increasing the strength of selection [20,40]. However, these studies and others compared expression responses of non-adapted individuals to adapted populations or selection lines [9,20,21,41,42], thereby examining patterns when divergence has already occurred. They provide little information on how evolutionary forces have acted in the past and shaped expression but show only the current state after divergence. Studying organisms that have been exposed to environmental change recently can give us more insight into the initial processes leading to divergence between populations experiencing novel environmental conditions and how changes in transcription may contribute to it. In particular, it is still unknown how short-term selection pressures are linked to long-term optimum expression levels. The two may differ because organisms may first activate stress responses that are beneficial and thus adaptive when they appear, but will not persist because costly to maintain on the long term [43,44], especially when they include negative stress effects like protein damage and the slowing down of cell cycle and protein synthesis [45,46].

The difference between short and long-term gene expression changes will depend on trade-offs between the benefits of immediate stress responses and their long-term costs. Adaptation necessitates optimal re-allocation of energy resources between maintenance and reproduction. Optimal solutions for this trade-off may differ between environmental stressors [47], which further complicates the study of plastic responses in gene expression and their fitness effects in variable environments. A beneficial response elicited by one environmental factor may be overridden by a negative effect in presence of a second factor and generate a pattern of maladaptive plasticity. Joint effects of stress factors can result in complex interactions and may not be simply deduced from single responses [48–50]. It is thus crucial to understand the trade-offs faced by organisms when adapting to changed environments [51,52]. Transcriptomics can give us insights into the mechanisms underlying trade-offs between responses to different stressors, and into energy allocation trade-offs between reproduction and maintenance within conditions. It can potentially show which physiological processes are activated, thereby giving us information about how resources are used. Trade-offs in stress responses can not only limit plastic responses but may also constrain evolution and future adaptation [51,53]. It is thus crucial to evaluate the adaptive value of observed plastic changes and to estimate how variation in gene expression levels is ultimately associated with fitness variation. In case plastic responses are adaptive and in the direction that should increase fitness (e.g., up-regulated genes under positive selection, down-regulated genes under negative selection), the evolutionary trait response should be in the same direction as the plastic response, a process sometimes referred to as the Baldwin effect [54,55]. Evolution may either shift the phenotypic mean in the same direction as the initial plastic response [56,57] or plasticity itself can be changed [9,57–59] and increase. In case of maladaptive plasticity, evolution should either result in a reduction of plasticity or in shifts of the mean opposite to the plastic response (i.e., counter-gradient selection [60], or genetic compensation [61]). Knowing the strength of selection acting on early-stage plastic responses can tell us more about the immediate adaptive value of plasticity and enable us to understand the evolution of plasticity.

In this study, we asked how *Tribolium castaneum* (the red flour beetle) was affected by heat and drought in single stressor treatments and in a combination treatment. *T*. *castaneum* is a globally distributed pest species of tropical origin [62]. Heat is an important factor for *Triboilium* as it is for many other insects [62]. Given that *T*. *castaneum* lives in dried food products, responses to low humidity might be of particular importance and the species is known to have specific adaptations to drought [63]. We combined a fitness assay with RNA-seq to measure gene expression and reproductive success in the same individuals. Observation of changes in gene expression allowed us to gain insights into the physiological processes affected by different stressors and identify potential resource allocation trade-off between reproduction and stress response. We could test whether the transcriptomic responses to heat and drought overlapped and were in the same direction. We further tested for interactive effects of the two stressors on expression changes in a combined hot-dry stress treatment. Since we measured expression and fitness in the same individuals with sufficient sample size, we were also able to estimate the transcriptome-wide distribution of selection intensities on gene expression levels giving us an unprecedented view of selection pressures on gene expression in different environments. With this data, we tested whether immediate plastic responses were adaptive or maladaptive in the new environments. We also estimated the selection acting on plasticity itself to understand whether selection on gene expression may result in indirect selection on plasticity. Overall, estimating the intensity of selection acting on variation in transcription levels allowed us to reach a better understanding of future adaptation and evolutionary gene expression changes in the stress treatments.

## Results

We used a *T*. *castaneum* strain (Cro1) [64], which was collected from a wild population in 2010 and adapted to standard control conditions (33°C, 70% relative humidity (r.h.)) since then. To assess the fitness and expression changes caused by stressful environmental conditions, we exposed the beetles to a drought, a heat, and a combined heat-drought treatment (conditions: Dry: 33°C, 30% r.h.; Hot: 37°C, 70% r.h; Hot-Dry: 37°C, 30% r.h.) We performed a fitness assay in all four conditions by measuring the number of adult offspring per female. We assessed gene expression changes relative to control conditions using whole transcriptome sequencing with RNA-seq performed on whole-body mRNA extraction. Individuals were transferred to treatments at the egg stage and stayed there during their whole lifetime. We measured fitness and expression in females at the age of eleven weeks. Because both measurements were performed in the same individuals, we could measure the direction and intensity of selection acting on gene expression levels in all four environments.

### Fitness assay

**Fig 1:**
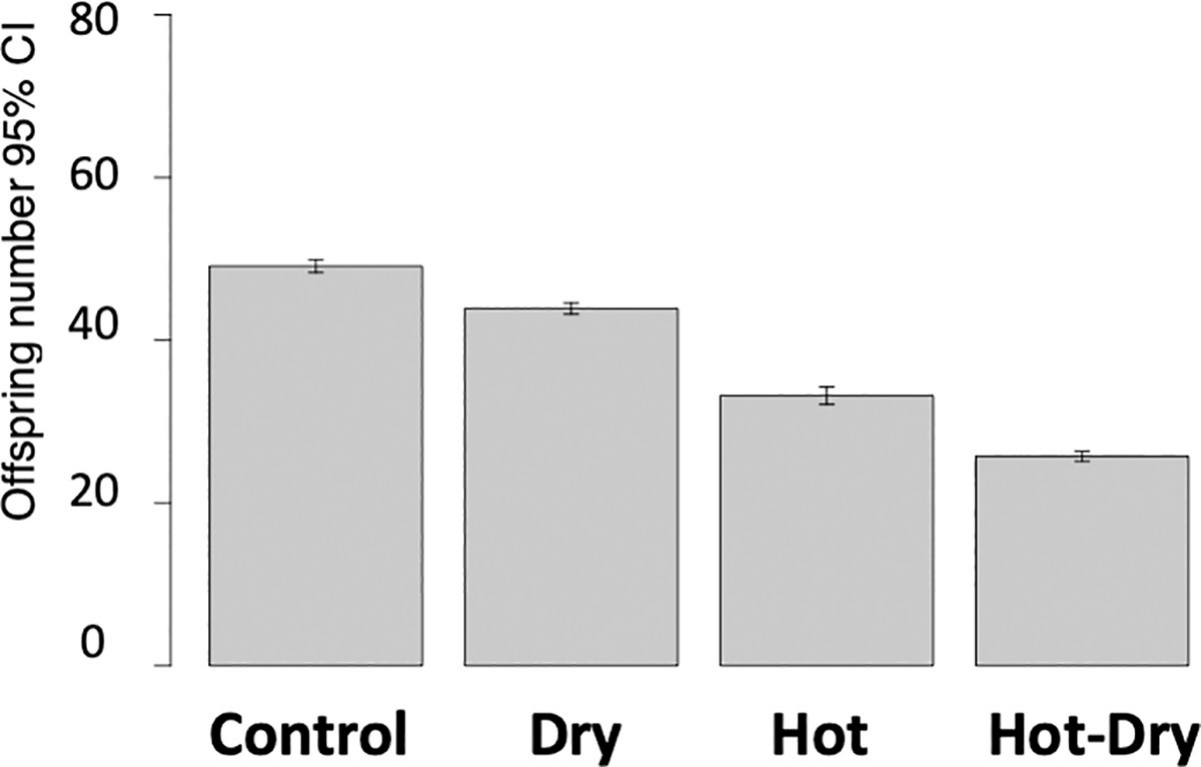
Offspring number of reproducing females in four different conditions: Control (33°C, 70% r. h.), Dry (33°C, 30% r. h.), Hot (37°C, 70% r. h.), Hot-Dry (37°C, 30% r. h.). Females could lay eggs for one week and adult offspring was counted five weeks later.

Offspring number of reproducing females decreased with increasing temperature (F_1,5157_ = 1981.07, P < 2.2e-16) and decreasing humidity (F_1,5184_ = 262.05, P < 2.2e-16), with a stronger effect of heat (-15.98 ± 0.57 SE) than of drought (-5.12 ± 0.50 SE). The lowest offspring number was found when heat and drought were combined (Fig 1, Table 1). Interaction between temperature and humidity was also significant (F_1,5128_ = 8.37, P = 0.003835) and led to an additional decrease of 2.22 ± 0.77 compared to purely additive effects. The proportion of reproducing females was significantly different between conditions (χ2 = 627.35, df = 3, P< 2.2e-16). The highest proportion of non-reproducing females was found in Hot (S1 Fig). Fitness data deposited in Dryad repository (https://doi.org/10.5061/dryad.gf1vhhmkn) [65].

**Table 1:** Results of the fitness assay. Number of adult offspring that females produced within one week of egg-laying in different conditions (Control: 33°C, 70% relative humidity (r.h.).; Dry: 33°C, 30% r.h.; Hot: 37°C, 70% r.h.; Hot-Dry: 37°C, 70% r.h.). For calculating offspring number per female only reproducing females were used.

Condition	N	Females with offspring	Offspring per reproducing female (±SE)	Variance offspring number per reproducing female
**Control**	1575	1514	49.09 ± 0.40	247.84
**Dry**	1642	1603	43.88 ± 0.36	204.24
**Hot**	1401	1005	33.18 ± 0.55	308.98
**Hot-Dry**	1567	1396	25.73 ± 0.31	136.76

### Gene expression response to heat is stronger than to drought

**Fig 2:**
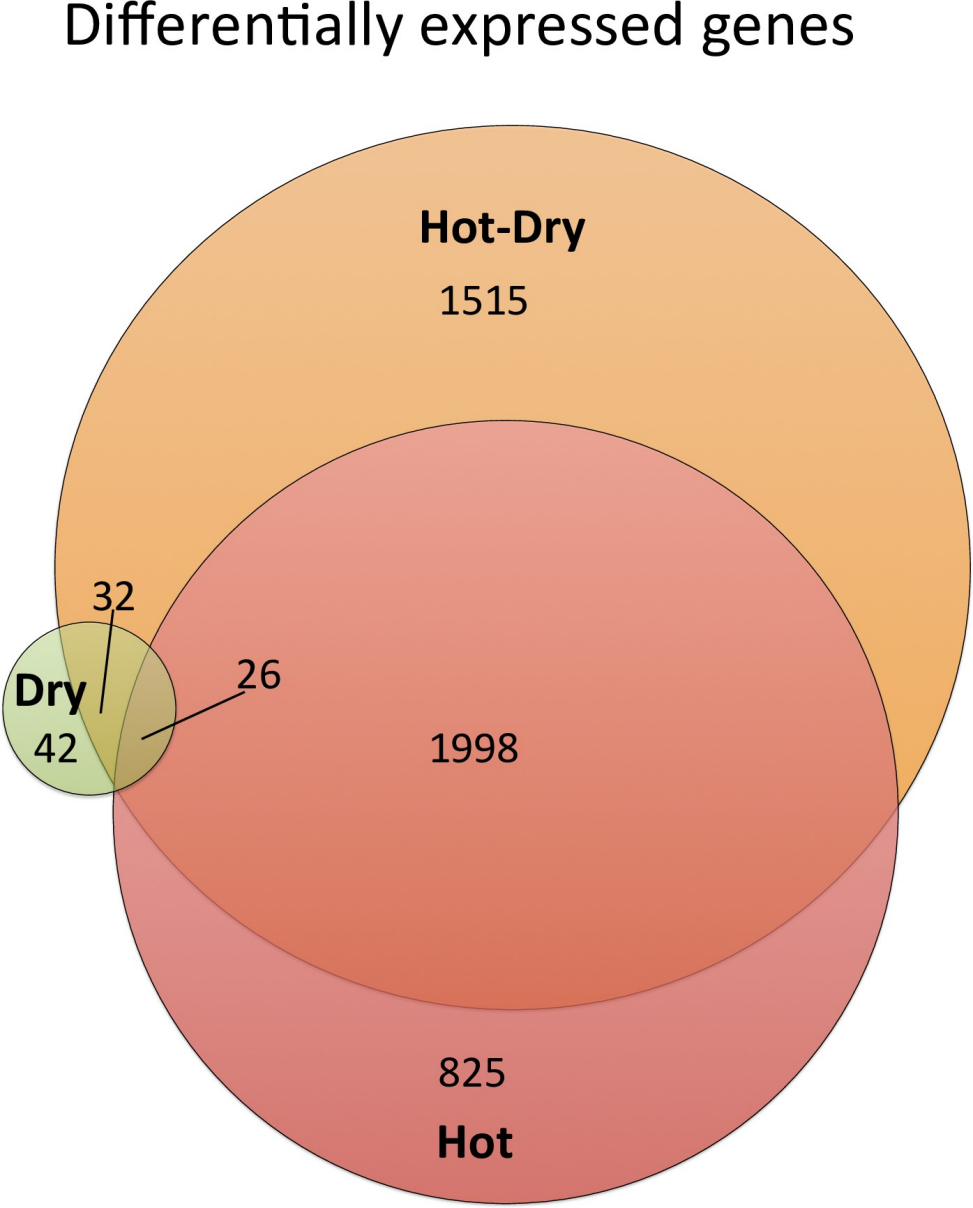
Venn-Diagram showing the number of differentially expressed genes in three treatments relative to control conditions. Overlapping regions represent genes that were found in more than one treatment and changed expression levels in the same direction. Sizes of circles as well as of overlapping regions are proportional to number of genes.

To evaluate the extent of the stress responses at the physiological and metabolic levels we assessed the changes of gene expression with a differential expression analysis (see Methods). The number of differentially expressed (DE) genes relative to Control was lowest in Dry and largest in Hot (Fig 2, Table 2, see also PCA plots in S1 Appendix). Drought induced up-regulation of 52 and down-regulation of 48 genes. In contrast, the response to heat showed a significantly higher number of DE genes than in Dry (up: 1594, down: 1255; permutation test *P* < 0.004). Overlap between heat and drought responses was significantly higher than expected by chance (χ2 = 17.75, d.f. = 1, p-value = 2.516e-05) and included 26 genes (Fig 2) with responses in the same direction (up: 25, down: 1) and 17 genes with responses in opposite direction. To investigate whether the DE genes were involved in specific biological processes, we performed pathway, protein domains, and Gene Ontology (GO) enrichment tests. Because of a low number of DE genes in Dry, only few enrichments could be detected in Dry (S2 Appendix, S3 Appendix), with up-regulated genes enriched in active ion transmembrane transporter activity (GO:0022853), and down-regulated genes enriched in hydrolase activity, hydrolyzing O-glycosyl compounds (GO:0004553), and protein family Thaumatin (IPR001938). Analysis of the *Tribolium* genome had revealed a high number of genes thought to be involved in endocrine regulation of diuresis, including several that encode putative neuroendocrine peptides like antidiuretic factors [66–69]. None of these genes was found to respond to the Dry treatment.

**Table 2:** Number of differently expressed genes (FDR < 5%) when comparing different conditions. Positive: higher expression in second condition. Negative: lower expression in second condition. Differential expression analysis was conducted in edgeR [70].

	positive	negative	total
**Dry vs Control**	52	48	100
**Hot vs Control**	1594	1255	2849
**Hot-Dry vs Control**	1866	1705	3571
**Hot vs Dry**	1553	1290	2843
**Hot-Dry vs Dry**	1928	1813	3741
**Hot-Dry vs Hot**	101	164	265

Genes up-regulated in Hot were enriched in many metabolic processes, e.g. carbohydrate metabolic process (GO:0005975), Citrate cycle (KEGG 00020), and Pyruvate metabolism (KEGG 00620) (S2 Appendix, S3 Appendix). The most strongly enriched category was chitin metabolic process (GO:0006030). A protein domain analysis also showed significant enrichment of heat shock proteins (IPR031107, IPR018181, IPR008978) (S3 Appendix). Down-regulated genes were enriched in pathways for DNA replication (KEGG 03030), nucleotide excision repair (KEGG 03420) and Ubiquitin mediated proteolysis (KEGG 04120) (S3 Appendix). The significant overlap between heat and drought response suggests that these genes are involved in a general stress response. However, no significant functional enrichment could be detected.

### Response to stressor combination is dominated by the heat response

When both stressors were combined in Hot-Dry, we found 3571 DE genes (up: 1866, down: 1705). Among them, 1515 (42.4%) were not found in single stressor treatments. However, only 69 of those genes (up: 30, down: 39) were found significantly DE between Hot-Dry and Dry, or Hot-Dry and Hot. This indicates that in most cases the combined stress did not induce expression changes in a different set of genes but modified their expression levels over and above their responses to single stressors. Compared to Hot, the Hot-Dry response had a significantly higher magnitude of expression change (permutation test: *P* < 0.0001), a higher number of down-regulated genes (*P* = 0.007), but a similar number of up-regulated and total number of DE genes (*P* = 0.29 and, *P* = 0.054, respectively). The functional response to Hot-Dry resembles the response to Hot, but more enriched GO categories and pathways could be found (S2 Appendix, S3 Appendix).

### Single stress responses are mainly not modified in combination

**Fig 3:**
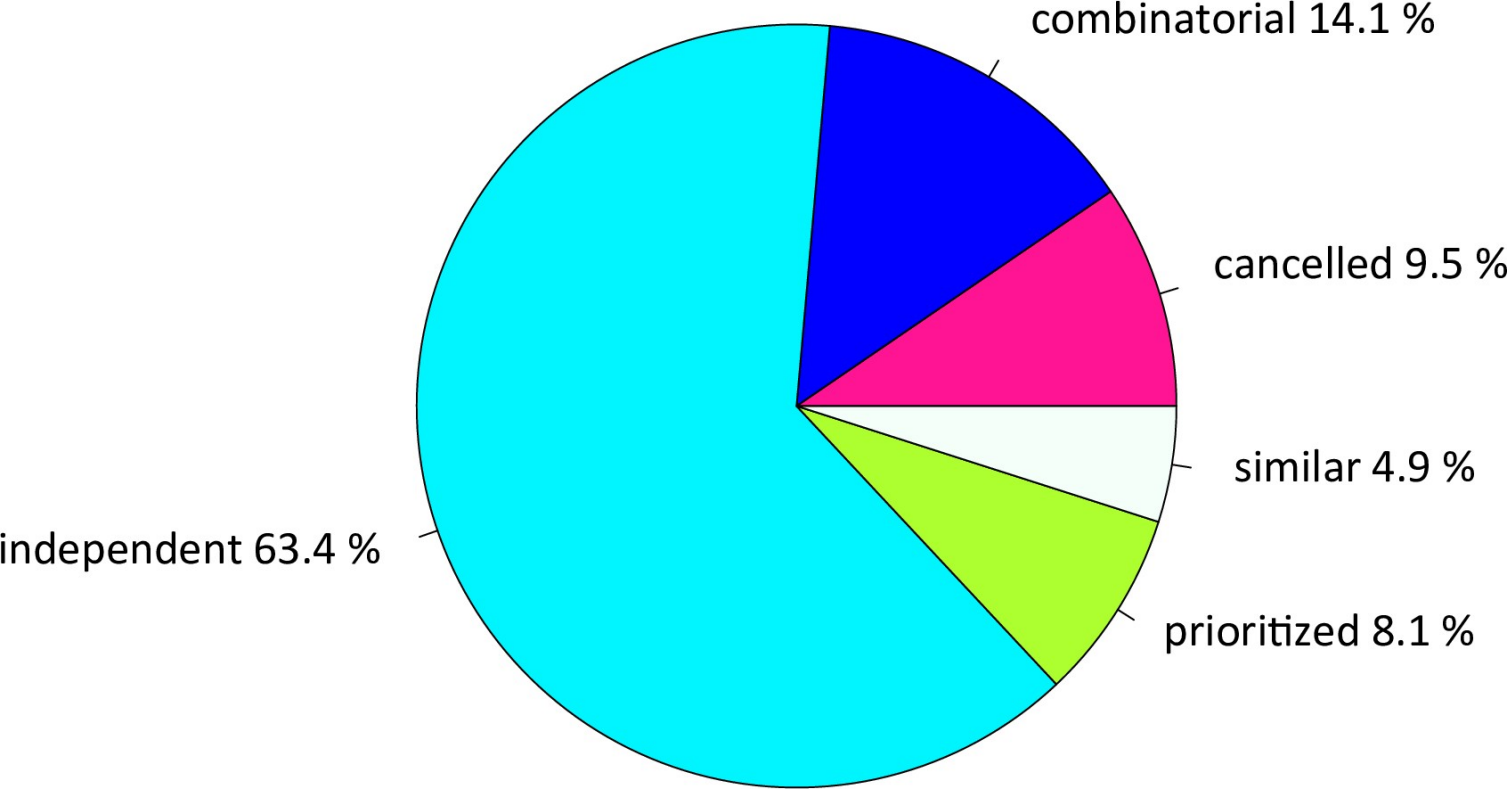
Response modes of the genes in Hot-Dry with a significant response to at least one of the treatments (Dry, Hot, Hot-Dry). *Combinatorial*: Similar levels in the two individual stresses but a different response to combined stresses; *cancelled*: transcript responses to either or both individual stresses returned to control levels; *prioritized*: opposing responses to the individual stresses and one stress response prioritized in response to combined stresses; *independent*: response to only one single stress and a similar response to combined stresses; *similar*: similar responses to both individual stresses and to combined stresses. Subcategories of different response modes, with more details about the most prevalent patterns, are given in S2 Fig.

To further examine how single stress responses are modified during combination, we classified the DE genes of all treatments into different response categories following [71] (see Methods and Fig 3). Only 5% of the genes showed a *similar* response mode, with same response to Dry, Hot and Hot-Dry (Fig 3). Most responding genes (63%) were classified as *independent*, with a response that is not altered in presence of a second stressor. Most of those genes showed the same response in Hot and Hot-Dry (60% of all genes, S2 Fig), but no response in Dry, in agreement with our DE analysis. 14% had a *combinatorial* response mode: They did not respond to heat and drought alone, but to their combination. These represent cases, in which presence of an additional stressor magnifies the effect of another. Interesting are genes with opposite responses to both stressors, but with one response *prioritized* when stressors occur simultaneously. These genes can be indicative of physiological trade-offs that constrain responses to stress combination. We found 8% of DE genes falling into that category. Most of them showed prioritization of the Hot response in Hot-Dry (7.5%, S2 Fig). This is in agreement with our DE analysis, which showed a high similarity between responses to Hot and Hot-Dry. 9.5% of expression responses were classified as *cancelled*, i.e., response disappears when another stressor is added. Most of these genes (6.7%) showed a significant response in Hot, but not in Dry and returned to control levels in Hot-Dry.

### Weighted coexpression network analysis

Instead of focusing on single genes, a gene co-expression analysis can provide additional insight into molecular mechanisms underlying trait variation. Genes do not act in isolation but are organized in pathways or functional networks with complex interactions [72,73]. Furthermore, considering modules instead of genes help to avoid the problem of multiple testing, since it reduces the high-dimensional data set to a few modules that are further tested for relationships with phenotypic traits. We used a weighted gene coexpression network analysis (WGCNA) [74] to identify modules of coexpressed genes. When conducted separately for each treatment, we detected very large modules in Control that were enriched for many GO categories like metabolic processes, signaling, and regulation, indicating that all these processes work in a coordinated way (S4 Appendix). In stress conditions, these large modules became separated into smaller networks according to the different functional processes that were previously linked in non-stressful conditions.

We also conducted a joint WGCNA using samples from all conditions together to infer to which extent functional modules were influenced by treatment conditions by testing for the association between each of the detected module’s eigengene (principial component of a module) and stress conditions (one-way ANOVA). We found that the three largest of the five detected modules did not show a significant association with condition (Table A in S4 Appendix). They probably represent groups of genes involved in homeostasis and maintenance of essential cellular functions that are independent of the stress condition the individuals experienced. In contrast, the two smaller modules showed a significant relationship with treatments. Next, we tested for an association between each module’s eigengene and fitness and how this was influenced by treatment condition (interaction between eigengene and condition in a two-way ANOVA with fitness as response variable). We found that the two modules significantly influenced by treatments were also strongly associated with fitness (S4 Appendix). We further found that the relationship between a module’s eigengene and fitness was significantly condition dependent in three of the five modules. However, since many genes lack functional annotation and the proportion of genes that were not assigned to any module was relatively high (39% in the joint analysis), information obtained by functional enrichment analysis of gene in a module remained limited.

Details of coexpression analysis and corresponding results and discussion can be found in S4 Appendix.

### Treatment effects on reproduction related processes

To get further insights into the molecular processes that link the observed decline in offspring number with transcriptomic data, we focused on genes and pathways known to be involved in egg production and in the mediators of the trade-off between stress response and reproduction [75]. We thus looked at the response of a gene set (S1 Table) made of juvenile hormone (JH), 20-hydroxecdysone (20 E), insulin/insulin-like peptides (IIS) target of rapamycin (TOR) signaling pathways (IIS-TOR), and vitellogenin, the main nutrient source of eggs, and vitellogenin receptors. We found that heat and heat-drought stress led to a significant down-regulation of all pathways and repression of vitellogenin and vitellogenin receptors (S2 Table). Drought did not show any significant effect. We selected a set of genes within these pathways with known effects on reproduction in *T*. *castaneum* [76–78]. A gene set test confirmed that these genes were mainly down-regulated in Hot and Hot-Dry (Table 3).

**Table 3:** Results of gene set enrichment analysis of genes involved in reproduction. Gene set enrichment test was conducted in edgeR using the *roast* function [70]. Prop.Down and Prop.Up give the proportion of genes that are down- and up-regulated. The direction of change is determined from the significance of changes in each direction and is shown in the Direction column. The *P*-value provides evidence for whether the majority of genes in the set are DE in the specified direction. The genes (N = 56) were selected based on [76–79].

Contrast	Prop. Down	Prop. Up	Direction	p-value
**Dry–Control**	0.054	0.036	Up	0.912
**Hot–Control**	0.464	0.071	Down	0.001
**Hot-Dry–Control**	0.589	0.071	Down	0.001

### Selection on expression levels is environment specific

Since we measured offspring number and transcription within the same individuals, we could estimate selection intensity on gene expression levels in each condition separately by performing a linear regression of relative fitness on standardized expression levels (z-score of read counts per million after TMM normalization). In control conditions, expression levels of 2179 genes showed a significant correlation with offspring number (negative: 2158, positive: 21, at 5% FDR). The two genes under strongest positive selection coded for vitellogenin (selection gradient ± SE: Vg1: 0.26 ± 0.05; Vg2: 0.24 ± 0.05). Another positively selected gene coded for a serine protease (TC000870) and is involved in oocyte development (GO:0048599). In Dry, Hot and Hot-Dry we could not detect any significant selection on gene expression levels after correcting p-values for multiple comparisons.

**Fig 4:**
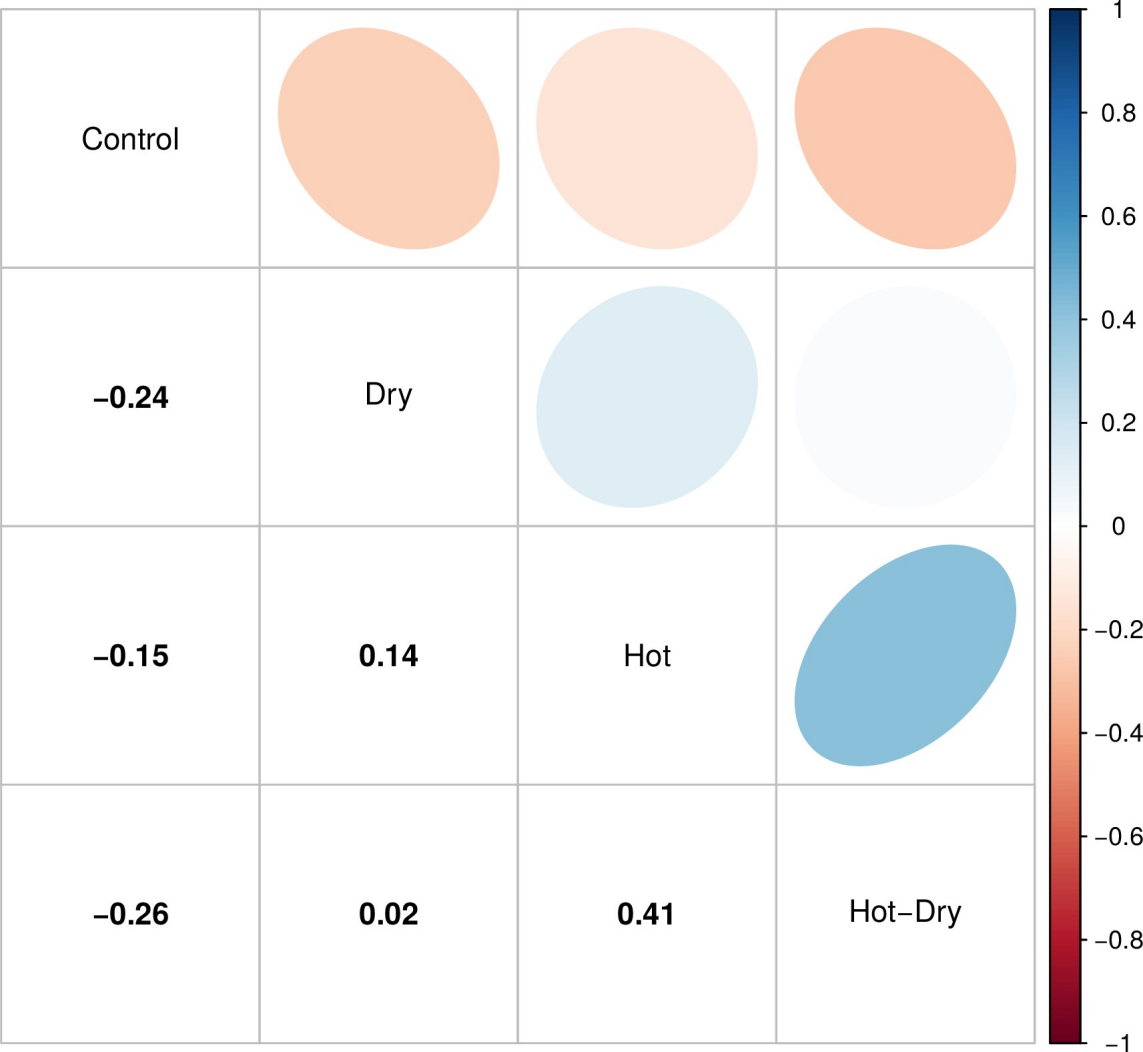
Pairwise correlations of selection intensities on single gene expression levels in different conditions. Blue indicates a positive and red a negative correlation. Values are given in the lower triangle. Confidence intervals for correlations: Control-Dry: -0.25, -0.22; Control-Hot: -0.11, -0.08; Control–Hot-Dry: -0.23, -0.20; Dry-Hot: 0.08, 0.11; Dry–Hot-Dry: 0.00, 0.03; Hot–Hot-Dry: 0.33, 0.36.

**Fig 5:**
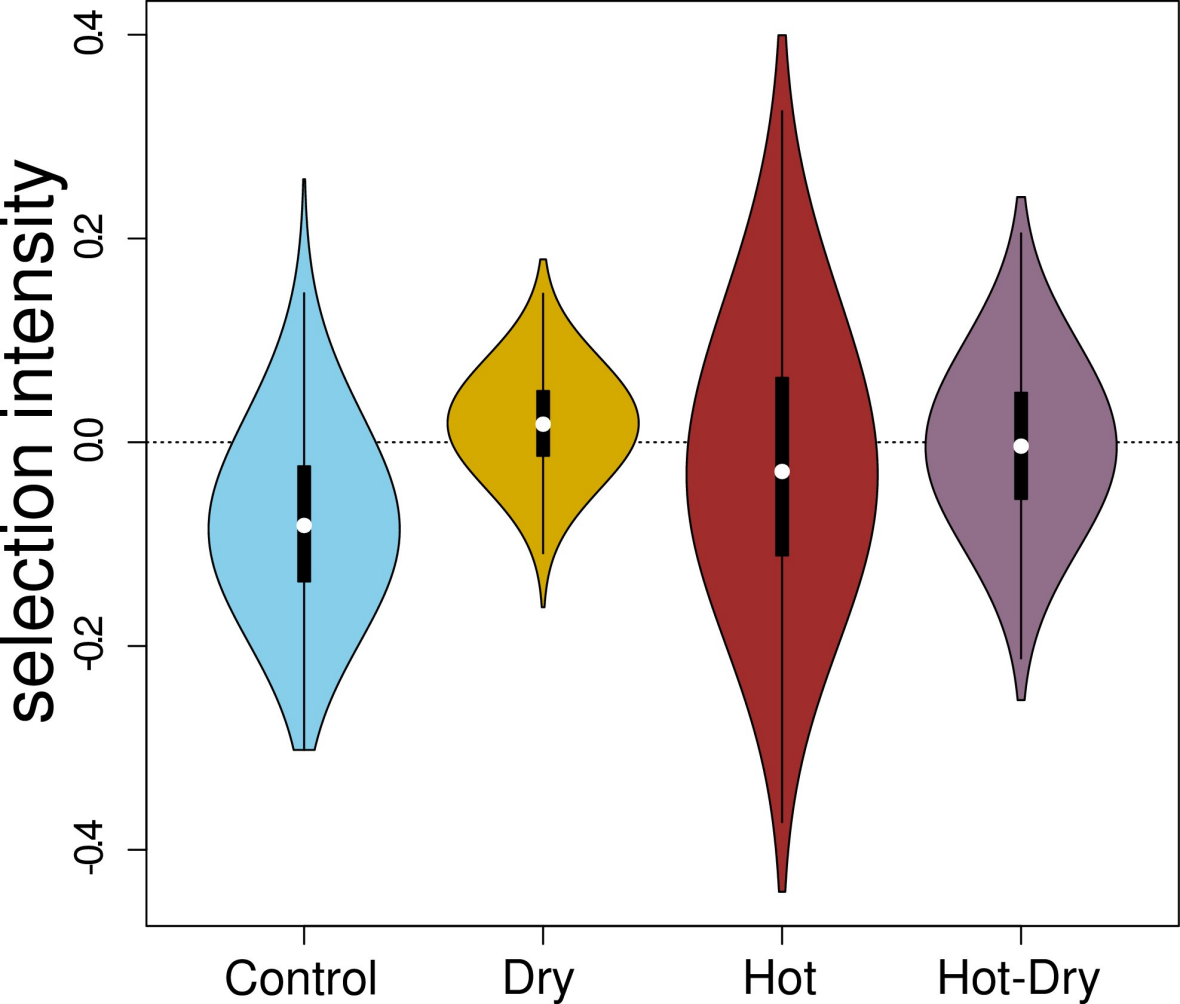
Distribution of selection intensities on gene expression levels under Control and treatment conditions. Each violin plot contains a boxplot of the data. White dots are medians and black rectangles represent inter-quartiles. The selection intensities were obtained as linear regression coefficients of relative fitness (number of adult offspring) on normalized RNA-seq read counts (z-score).

To compare selection acting on expression levels under different conditions and avoid stringent significance thresholds on single-gene fitness-expression correlations, we then estimated the correlation of selection intensities among treatments. We found significant negative correlations of selection intensities between Control and all stress treatments, and positive correlations among stress treatments (p-values < 2.2e-16) (Fig 4). Control and Dry had the strongest negative correlation (-0.24), while Hot and Hot-Dry had the highest positive correlation (0.34). Furthermore, significantly DE genes responding to Hot and Hot-Dry were over represented among those that were negatively selected in control conditions (Hot: χ2 = 158.62, df = 1, p-value < 2.2e-16, Hot-Dry: χ2 = 177.97, df = 1, p-value < 2.2e-16), with 361 (22.6%) up-regulated genes in Hot and 417 (22.3%) in Hot-Dry. The magnitudes of selection intensities were also significantly different between conditions (median (and SD) of absolute values in Control: 0.09 (0.06), Dry: 0.03 (0.03), Hot: 0.09 (0.07), Hot-Dry: 0.05 (0.04); Kruskal-Wallis rank sum test: χ2 = 9333.3, df = 3, p-value < 2.2e-16), with the majority of genes negatively selected in Control, and similar proportions of genes under positive and negative selection in stress treatments (Fig 5). The Dry treatment had the largest negative correlation of expression levels with Control and the largest number of genes switching sign relative to Control (9591). However, the magnitude of change in selection intensity of those genes was lowest in Dry (median: 0.14). In contrast, the Hot treatment had the strongest magnitude of change in selection intensity compared to Control (median: 0.19, 6644 genes), followed by Hot-Dry (median: 0.16, 7412 genes). Overall, a large majority of genes switched from negative to positive selection (Dry: 0.86, Hot: 0.76, Hot-Dry: 0.8).

### The response in gene expression is mainly adaptive

**Fig 6:**
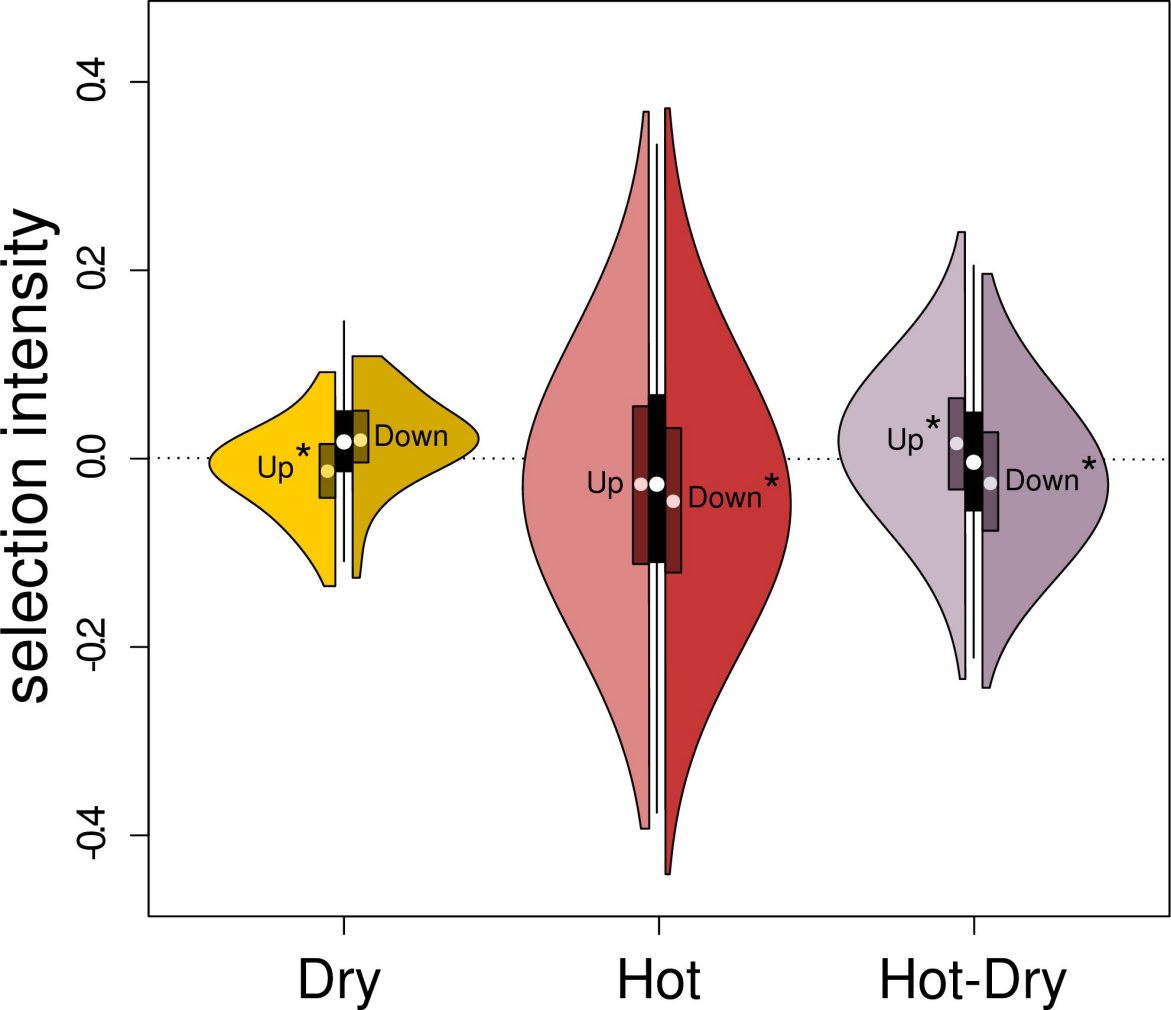
Selection intensities on expression levels of genes that showed significant responses to stress treatments (DE genes). The left half of each violin plot is the distribution of selection intensities of up-regulated genes, relative to Control, while the right half is for down-regulated genes. The median and inter-quartile range are represented as a white dot and a dark rectangle, respectively. The central boxplot represents variation of selection intensities of genes whose expression was not significantly different from expression in Control (at 5% FDR). Significance of the shift in selection intensities of the up- and down-regulated DE genes relative to the non-DE genes is marked with the star symbol (*). Significance was determined with a permutation test with 10,000 permutations (see **Methods**).

To examine whether the plastic responses in gene expression are adaptive, we tested if significantly up-regulated genes were under more positive and significantly down-regulated genes under more negative selection than non-responding genes. We found that the response to Hot-Dry was mainly adaptive: Down-regulated genes were under significantly more negative selection and up-regulated genes under more positive selection compared to non-responding genes, respectively (permutation tests: *P* < 0.0001) (Fig 6). In contrast, some parts of the response in Dry seemed maladaptive: Down-regulated genes were not under significantly different selection, but up-regulated genes were more negatively selected (Fig 6). In Hot, the response was partly adaptive since down-regulated genes were significantly more negatively selected, while up-regulated genes were not under significantly different selection (Fig 6).

### Indirect selection on plasticity from selection on gene expression

We were interested to examine whether selection on expression levels in the treatment might also potentially influence plasticity of the respective genes during future adaptation. First, we tested for an association between expression levels in the treatments and the strength of their plastic response. Since the individuals used in this study were members of full-sib families that were split across conditions (see Material and Methods), we could use the differences in family means between conditions as an estimate for the plasticity of a certain gene and then correlate this with its expression level (family mean) in the treatment. We found a positive correlation between expression levels in the treatment and plastic changes (mean/median of correlations: Dry: 0.48/0.51, P < 0.0001 (10,000 permutations); Hot: 0.60/0.65, P < 0.001; Hot-Dry: 0.57/0.61, P < 0.001). Highly expressed genes generally showed a strong up-regulation in response to the treatment (and lowly-expressed genes showed a strong down-regulation). This suggests that selecting for high or low expression could indirectly influence plasticity. Second, we tested whether our previous estimates for selection on expression in the treatments were associated with indirect selection on plasticity. To estimate selection on plasticity, we used the correlation between plasticity (differences in family means between conditions) and the mean family fitness in the treatment. If, for instance, a family shows a large increase in expression of a certain gene and a high fitness in the treatment, the plasticity of that gene would be under positive selection. We found that selection on expression levels in the treatments were generally correlated with selection on plasticity (Dry: 0.36, *P* < 0.001 (10,000 permutations); Hot: 0.24, *P* < 0.001; Hot-Dry: 0.62, P < 0.001, S3 Fig). This suggests that selection on expression levels in the stress treatments may result in indirect selection on plasticity. For instance, when adaptive, plasticity may increase in future generations. Alternatively, plasticity may decrease when maladaptive because under more negative selection, as suggested by the results in the Dry treatment (Fig 5).

## Discussion

### Selection on gene expression

By measuring the relative abundance of transcripts and one component of fitness within the same individuals, we could assess the strength of selection acting on gene expression variation within each environment. The distribution of selection intensities, measuring the phenotypic association between fitness and gene expression [80,81], was informative of the strength and direction of net selection in each environment. Net selection includes direct selection on a gene’s expression and indirect selection from correlated expression changes at other genes (see below). Under benign control conditions, the distribution of selection intensities had the most negative mean and median with a small proportion of transcripts under significantly positive selection. Among them, vitellogenin genes and few other reproduction-related genes were under strongest positive selection. Vitellogenin genes (Vg1, Vg2) are directly involved in egg production [82]. Their strong positive association with offspring number suggests that variation in this component of fitness is mainly driven by differences in egg production among individuals. We could further confirm that variation in offspring number was mainly explained by variation in fecundity and not variation in larvae or pupae survival (see S5 Appendix). This shows that our experimental design was able to capture meaningful associations between gene expression in female individuals and their reproductive output.

The small proportion of transcripts under positive selection in Control suggests that the number of processes positively associated with offspring number is small when conditions are benign. Under such conditions, processes not directly contributing to fitness should be repressed to allow investing most resources into reproduction. Shifts in temperature and humidity then caused an increase of the proportion of gene expression variation under positive selection, with higher median and mean selection intensities in the three stress treatments. The changes of the direction of selection, from mostly negative to more positive, affected both the genes that significantly changed their expression plastically (DE genes), and those that did not. This suggests that under stressful conditions, processes unrelated to egg production and fecundity affected fitness positively. Accordingly, genes involved in heat-stress protection (e.g. heat shock proteins Hsp 68, Hsp23) and many metabolic processes (e.g. tricarboxylic acid cycle GO:0006099, aerobic respiration GO:0009060) were up-regulated and under positive selection in Hot-Dry (see S2 Appendix). The concomitant down-regulation of genes involved in cell growth, cell cycle (e.g. DNA replication KEGG03030, S3 Appendix), and reproduction is also typical of stress responses [30,83,84]. Similarly, JH genes and genes in the IIS-Tor and 20E signaling pathways were down-regulated in Hot and Hot-Dry. They are known to be involved in the trade-off between stress-responses and cellular growth and maintenance [75,85,86]. Selection on down-regulated genes was mostly negative in Hot and Hot-Dry, and thus adaptive. Regardless, down regulation of genes involved in cell growth and reproduction should result in decreased fecundity. As a result, we observed a general decrease of average fitness in the stress treatments. Reduction in fecundity was also likely affected by the expression of heat shock proteins, whose fitness costs are known [84,87,88]. This together with the large switch of 80% of the genes from negative selection in Control to positive selection in the treatments, suggests a general re-channeling of resources from growth and reproduction to stress response and protective processes.

Combining results of DE and network analysis by overlaying DE and functional modules revealed that large parts of Control modules that are related to metabolism were conserved across treatments. Their genes did not show a significant change in expression and were involved in processes that are required for homeostasis. Other parts of the modules stayed closely connected, but many genes including the modules’ hub genes showed a response to stress, thereby partly rewiring functional networks. Stress treatments seem to disrupt the normally tight connections between metabolism and replication. In a joint network analysis with samples from all conditions we detected five modules and found that associations of three of these modules with fitness were significantly condition dependent. The WGCNA analyses thus confirm that gene expression and its underlying network structure experienced different selection pressures in the treatments.

Variance in selection intensities also varied greatly between treatments, with lowest variances in the dry environments (Dry and Hot-Dry) and largest in the Hot treatment. Reduced variation and a mean selection intensity close to zero as in Dry can be associated with stabilizing selection and expression levels closer to their optimum. *T*. *castaneum* might thus be better adapted to dry than humid conditions. In fact, *T*. *castaneum* is known to have special anatomical adaptations to cope with extremely dry conditions [89]. This points to a possible long evolutionary history of encountering drought and strong past selection for drought resistance. Induction of preexisting drought response mechanisms may have helped keep the physiological and metabolic responses in check and limit non-optimal gene expression levels and reduce the variation in fitness among individuals. The observed maladaptive plastic responses in Dry can also be interpreted in that context, where too large responses of over expressed genes may have overshot their optimal response and led to negative selection intensities.

In contrast, the variance of selection intensities was especially high in Hot (Fig 6). Hot was also the condition with the highest proportion of non-reproducing females. We may speculate that this condition was exceptional to *T*. *castaneum* and was not often encountered in the past. Previous selection on expression imposed by a combination of high temperature and high humidity may have been low. We found evidence of an increase in the variance of expression levels in the stress treatments. The coefficients of variation for gene expression levels in Hot were higher than in Control (S4 Fig). Exposure to a strong stress for which no adequate response evolved previously might have led to disruption of homeostasis and expression of hidden genetic and phenotypic variation [90,91].

### No physiological trade-offs between single stressors

The transcriptomic responses to heat and drought were very contrasted with a small but significant overlap. Only a minority of overlapping genes (17 out of 43) showed a trade-off in expression between the two conditions, and no functional enrichment could be detected. Different physiological processes are thus likely affected by the two stresses. The lack of a strong trade-off between the individual stress responses was also evident when investigating the combined transcriptomic response in the Hot-Dry treatment. Most DE genes in Hot-Dry responded to a single stressor independently of the presence of the second stressor (Fig 3). Such an additive combined stress response can be expected when individual stressors require different protection mechanisms and affect different pathways, and are thus likely not interfering with one another [92].

Furthermore, as expected from the single stress responses, the combined response was dominated by the heat response. This is in agreement with many studies showing that heat is a major driver of expression change, especially in ectotherms [9,93–96]. The combined effect of heat and drought on fitness was close to the combined reduction of fitness in Hot and Dry. However, due to our large sample size, we detected a significant interaction between heat and drought on offspring number, but with a small effect size (-2.22 ± 0.77). Even when there are no opposite physiological effects, stress response mechanisms are accompanied with costs because all of them rely on the same pool of limited resources. Additive effects might therefore only be observed until a certain threshold of resource consumption is reached [51,97]. When an individual is close to its maximum capacity of stress tolerance, addition of a stressor that has only small effects when applied singularly can lead to strong detrimental consequences [98]. It might be that our beetles were close to that threshold where competing energy demands for different stress responses can lead to synergistic effects. The addition of a drought stress to the heat stress apparently emphasized the effect of the heat stress, as seen in the transcriptomic response where the Hot-Dry response mainly differed in magnitude compared to the Hot response.

### Adaptive value of plasticity and indications for evolutionary adaptation

Our study provides estimates of the strength of natural selection acting on gene expression levels and can thus give indications for future evolutionary changes in trait values and in plasticity. Maladaptive plasticity can promote trait evolution by increasing the strength of selection [40,99] and lead to a reversal of the ancestral plastic response during long-term evolutionary adaptation [19,20,26,100]. Adaptive plastic changes can become fixed differences when plasticity is lost because costly to maintain, leading to genetic assimilation [27,47,101]. Alternatively, evolution can increase the magnitude of plastic responses if they are adaptive. Both adaptive and maladaptive plasticity can thus contribute to evolutionary divergence, but the adaptiveness of a plastic response determines its contribution to future divergence and evolutionary trajectories.

Our results suggest that plastic responses in Hot-Dry and down-regulation in Hot were mainly adaptive to exposures to thermal and humidity stresses. For a majority of genes, relative mRNA abundance was changed in the direction favored by selection. Knowing the selection intensities on mRNA abundance, it is obviously tempting to speculate on possible evolutionary changes in expression levels, and in their plasticity. Some of the adaptive trait changes observed may persist over evolutionary times without genetic changes if plasticity itself is not too costly, leading to phenotypic divergence but no genetic divergence relative to ancestral conditions. As suggested by Ghalambor et al. [40] genes with maladaptive plastic responses might be those that will show the strongest changes during evolution. Furthermore, the positive correlation between expression levels in the treatments and their degree of plasticity suggests that selection on gene expression in the treatments should result in correlated changes in their plasticity. However, immediate stress responses might not remain beneficial during long-term adaptation to constant high temperature because they trade off with reproduction. Therefore, adaptive but costly plastic responses may become maladaptive over time and be reversed despite the immediate benefits provided by their protective functions. An example might be heat shock proteins, which were among the most strongly responding genes in this experiment and mostly under positive selection in Hot-Dry. Their immediate protective functions are well known [102], as well as the reproductive costs of their over-expression [88]. Although initially adaptive, some of the plastic expression changes can be reversed in future evolved populations when other resistance mechanisms may arise (e.g. enzymes, which are more stable at higher temperatures), making costly stress protection expendable. Similarly, down-regulation of reproduction-related genes may not persist. Evolutionary changes resolving the trade-off imposed by stress responses will be favored and drive adaptive differentiation relative to the ancestral condition. Immediate plastic responses may then look as if maladaptive when compared to evolved trait divergence, a pattern often found in empirical studies [100].

Taken together, our results show a mix of responses sometimes in the direction of natural selection and sometimes in an opposite direction. Overall, we expect long-term evolution to increase differentiation relative to the ancestral condition (here Control) in genes whose response will remain adaptive, but to decrease differentiation in those genes involved in strong fitness trade-offs. Decreased differentiation of costly plastic responses may cause later inferences of the adaptive value of plasticity biased towards findings of maladaptive plasticity, although immediate responses to environmental changes may have been adaptive and helped populations to persist. We should thus be cautious when interpreting the results of comparisons between evolved populations and ancestral plastic responses. Those studies may, however, help untangle the genes involved in plastic responses from those responsible for adaptive divergence [9,19]). Our study was not well armed to answer that question but showed the adaptive value of immediate plastic changes more clearly than previous studies.

There are two major caveats to keep in mind when interpreting selection gradients. First, a selection gradient does not imply direct causation between relative mRNA abundance levels and offspring number, in any case. Second, our estimates of phenotypic selection include both direct selection acting on expression levels and indirect selection caused by changes at correlated gene expression levels. Indirect selection can strongly affect the evolutionary trajectory of a trait via selection on genetically correlated traits [80,103]. Selection gradients, or intensities cannot be used to identify the direct targets of selection. Traits also change by indirect selection acting on genetically correlated traits [104]. Given that genes do not act in isolation, it is likely that indirect selection on expression of interacting genes play an important role [7]. Future evolutionary changes in the three treatments also depend on the existence of additive genetic variation in expression and genetic correlation with fitness [105–107]. While we have estimated the direction and strength of phenotypic selection acting on gene expression, we cannot predict evolutionary changes because we did not attempt to estimate the additive genetic co-variance with fitness of the traits under selection. Future work based on long-term evolution can address whether those selection pressures translated into corresponding evolutionary changes.

Nonetheless, our results constitute a resource to better understand the physiological and metabolic processes involved in the adaptation to the two different stressors and their combination. For instance, in the most stressful treatment, Hot-Dry, up-regulated genes under positive selection were enriched in many metabolic processes (e.g. aerobic respiration, citrate metabolic process), while down-regulated genes under negative selection showed enrichment in negative regulation of metabolic processes (GO:0009892). Thus, a part of the adaptive plastic response resulted in enhancement of metabolic activity, potentially improving females' reproductive output. However, because correlative, the selection intensities can only point to candidates genes whose effects on fitness would need to be further functionally validated.

One drawback of our study is the exclusion of males from the gene expression measurements. We decided to focus on females, because they have to invest more into reproduction compared to males. Energy allocation trade-offs between responses to different stressors, as well as between stress protection and reproduction should be more pronounced here. We could also show that number of adult offspring was mainly dependent on egg number (S5 Appendix), suggesting a minor contribution of males to fitness. Consequently, it is likely that correlation of male expression with reproductive output would be low.

## Conclusions

Our approach shows how transcriptomics can be used to get information about the relative importance of different stressors, their interaction, and the potential constraints acting on plastic and evolutionary responses when several environmental variables change at the same time. We were thus able to evaluate the immediate adaptive value of the plastic changes in gene expression. By adaptive, we here meant immediate fitness increases associated with changes in gene expression. Therefore, our study strongly contributes to our understanding of how plasticity may affect fitness at the early onset of adaptive divergence and gives indications of potential future changes in gene expression and its plasticity. It shows that some parts of the plastic response are adaptive, whereas others are maladaptive, potentially also leading to the correlated evolution of the plasticity of the responding genes. However, further work is needed to clarify how we can use plastic responses to predict long-term evolutionary outcomes, for instance by using long-term evolution experiments.

## Materials and methods

### Animal rearing and stress treatments

We used the *Tribolium castaneum* Cro1 strain [64], collected from a wild population in 2010 and adapted to lab standard conditions (33°C, 70% relative humidity) for more than 20 generations. Beetles were kept in 24h dark on organic wheat flour mixed with 10% organic baker's yeast. We sterilized the flour and yeast by heating them for 12h at 80°C before use. We tested the response of fitness and gene expression of the beetles to heat, drought, and a combination of both stressors. The conditions in the treatments were: Hot: 37°C and 70% r. h., Dry: 33°C and 30% r. h., Hot-Dry: 37°C and 30% r. h. Parents of the experimental beetles were reared and mated in control conditions at the age of four weeks in 15 mL tubes with 1 g of medium. Each virgin male was mated with a virgin female. After four days, in which the beetles could mate and lay eggs, each mating pair was transferred to a new vial. We repeated this three times, resulting in four vials per mating pair containing medium and eggs. Vials of each mating pair were randomly assigned to the four different conditions, resulting in full-sib families split across all conditions. Male and female offspring (four females and four males per family and condition) were separated at the pupal stage and transferred to 10 mL tubes with 1 g of medium and remained there until they were used for the fitness assay eight weeks later. After the fitness assay, males and females were transferred to 1 mL tubes, frozen in liquid nitrogen and stored at -80°C. We made sure that all beetles were alive before they were snap-frozen. The fitness assay was started in the morning and stopped in the afternoon one week later by removing the mating pair, which was then immediately frozen. Beetles should not show a diurnal cycle since they were kept in 24h dark.

### Fitness assay

To test the effects of the different conditions on fitness, we measured reproduction in 6183 virgin females (ca. 1500 per condition, Table 1). We mated each virgin female with one unrelated male from the same condition in 15 mL tube with 1 g medium. The male was removed after 24 h. Females were removed from the tubes after one week of egg laying, and 9 g medium was added to provide food for the developing offspring. After five weeks the number of offspring was counted. At this time, all offspring had reached the adult stage. Some females did not produce any offspring, in proportions that differed between conditions. To test whether there was an effect of treatment on the number of reproducing and non-reproducing females, we used a generalized linear mixed model with reproduction success (binomial: offspring/no offspring) as response and condition as fixed effect. Since some of the tested females and males were full-sibs and developed within the same tube, we used male and female families as random factors to account for non-independence due to relatedness and a shared environment during development. To test how offspring number of reproducing females was influenced by conditions we used a linear mixed model with offspring number as response, temperature, humidity and their interaction as fixed effects and female and male family as random factors. Denominator degrees of freedom were estimated using Satterthwaite approximation. Statistical analyses were performed using the Lme4 package [108] version 1.1–17 in R [109].

### RNA extraction, library preparation and sequencing

183 female beetles with known fitness (ca. 45 per condition), which had been stored at -80°C, were homogenized in Tri-Reagent^®^ (Zymo Research, California, USA) using an electric bead mill. RNA was extracted with the RNA Mini Prep kit (Zimo Research, California, USA) following the instructions of the manufacturer. RNA-quality was checked with the Bioanalyzer 2100 (Agilent, Waldbronn, Germany). Only RNA samples with a RIN value > 9 were used. Concentrations were measured with aQubit^®^ Fluorometer (Life Technologies, California, USA). Libraries were created with 500 ng RNA for each individual separately with the LEXOGEN mRNA-Seq Library Kit following the manual (LEXOGEN GmbH, Vienne, Austria). Library quality was checked on a TapeStation (Agilent, Waldbronn, Germany) to make sure that they were not affected by primer dimers or overcycling. Concentrations were determined by qPCR. Libraries were diluted to the same molarity. Concentrations of dilutions were checked again by qPCR and libraries were pooled (36 libraries per pool). All treatments were randomized during RNA-extraction, library preparation, and sequencing. The single-end sequencing was performed in five runs on the Illumina NextSeq 500 (Illumina, Inc, California, USA) using the 75 cycles High Output Kit. Each run resulted in 550–600 million reads that passed the internal sequencer filter. On average we obtained 14874063 reads per sample with an average quality of 33.2 (Phred score). After quality control using FastQC (www.bioinformatics.bbsrc.ac.uk/projects/fastqc), reads were mapped the reference genome (ftp://ftp.ensemblgenomes.org/pub/release30/metazoa/gtf/tribolium_castaneum/Tribolium_castaneum.Tcas3.30.gtf.gz) with STAR v.2.5 [110] (adaptors were trimmed and the first 10 bases were hard trimmed, minimum average quality Q10, minimum tail quality 10, minimum read length 20). We then used FeatureCounts [111] to count the number of reads that mapped to each gene in the reference genome. On average, 86.7% of the reads mapped to a unique position and we obtained on average 9205466 reads per sample for producing count data. Mapping as well as read counting was performed within the data analysis framework SUSHI [112]. Exact numbers of reads for each sample, their mean quality, and number of reads that were finally used for producing the count data for further analyses can be found in S3 Table.

### Differential expression and enrichment analysis

We conducted a differential expression (DE) analysis using the R package edgeR [70]. We tested for differently expressed genes between the treatments (Dry, Hot, Hot-Dry) relative to the control as well as to each other. A gene is classified as DE with a FDR ≤5% after adjusting for multiple testing [113]. Additionaly, we conducted a differential expression analysis using DeSeq2 [114] to confirm that our results were robust and not dependent on the program used for DE analysis. The results were consistent with the edgeR analysis (see S1 Appendix): We obtained very similar number of DE genes and identified mainly the same genes. We also checked the distribution of p-values for differential expression (S1 Appendix). We found that in all conditions the distribution was uniform with a clear peak close to zero, thus confirming that there were indeed true positives in our data that could be identified by false discovery correction. To test whether the number of DE genes (relative to Control) was significantly different between two environmental conditions a permutation tests was used. For each permutation entire RNA-seq samples of the two groups were randomly assigned to conditions and the edgeR analysis repeated. Significance was assessed by number of times the observed DE number was higher than the DE number obtained by permutations. To test whether the magnitude of change in expression levels relative to control was significantly different between Hot and Hot-Dry, we performed a permutation test. Absolute log2-fold changes of each transcript were randomly assigned to the two groups and differences in the mean were calculated. We then compared the distribution of differences obtained by permutations to the observed difference between mean absolute log2-fold changes in expression. Gene set enrichment analyses for immune response genes and reproduction related genes were conducted in edgeR using the *roast* function [115]. The significance cutoff for genes contributing to the proportion of down-regulated genes is z < -√2 and z > √2 for proportion of up-regulated genes [70]. A GO enrichment analysis of DE genes was performed with gProfiler Version: r1622_e84_eg31 [116] and pathway and protein domain enrichment analysis with STRING v.10.0 [117].

### Classification of response mode

Following [71] we created 20 predefined expression profiles each representing a potential response when two single stressors are combined: *Combinatorial*: similar expression levels in single stress treatments but a different level in stress combination, *cancelled*: response to one or both single stressors but expression levels similar to control conditions when both are combined, *prioritized*: opposite responses to single stressors and expression levels in combination similar to one of them, *independent*: response to only one single stressor and the same response in combination, *similar*: same response in each of the two single stressor treatments, and combination. For creating predefined expression profiles we used 0 as control level, 1 and -1 as expression levels for up- and down-regulation, e.g. expression profile for an independent response could be: CT:0, D:0, H:1, HD:1. We then created a dataset consisting of all genes showing a significant response in at least one treatment (4419 genes in total). Correlation between normalized read counts (cpm, TMM normalization) of these genes and each of the predefined expression profiles was tested and genes were assigned to the category with the highest correlation.

### Selection

We measured selection intensity on gene expression separately for each treatment using univariate linear regression methods [80,118]. Fitness (number of adult offspring) was normalized by dividing each individual value by the mean (w’ = w_i_/mean(w)). For each gene, expression levels were first normalized to cpm (counts per million, TMM normalization) using edgeR and then transformed to standardized z-scores by subtracting the mean and dividing by the standard deviation (z = (x_i_- mean(x))/ SD(x)). Resulting regression coefficients of relative fitness on standardized expression levels give an estimate of the selection intensity. P-values were corrected for multiple comparisons. To test whether up- and down-regulated genes were under significantly different selection compared to genes without a significant response, we used permutation tests. For each permutation (10,000 for each test) we randomly assigned the categories “not DE” and “up” (or “down” respectively) to each estimated selection intensity and calculated the difference in mean selection intensity between both groups. Significance was tested by counting the number of permutations that showed a difference higher or equal to the observed one. To confirm significance of the correlation between selection intensity on expression levels in the treatment and selection on plasticity of the respective genes we used permutation tests (10,000 tests). We randomly sampled selection intensity on expression levels and assigned them to estimated selection on plasticity and calculated the correlation again. Proportions of permutations that exceeded the observed correlation give the respective p-value.

## Supporting information

S1 Appendix Comparison gene expression analysis with edgeR and Deseq2. (PDF)

S2 Appendix Results of GO enrichment analysis of DE genes. (XLSX)

S3 Appendix Enriched pathways and protein domains of DE genes. (XLSX)

S4 Appendix Weighted gene coexpression analysis. (PDF)

S5 Appendix Fecundity assay: Description and results of fecundity assay testing treatment effects on egg number, hatching rate, and larvae survival. (PDF)

S1 Fig Proportion of reproducing females in four different conditions. Control: 33°C, 70% relative humidity, N = 1575; Dry: 33°C, 30% r.h., N = 1642; Hot: 37°C, 70% r.h., N = 1401; Hot-Dry: 37°C, 30% r.h., N = 1567.
(PDF)

S2 Fig Subcategories of different response modes giving more details about the most prevalent patterns: Response modes of significantly responding transcripts in the stress treatments (Dry (D), Hot (H), Hot-Dry (HD). Combinatorial: Similar levels in the two in the two individual stresses but a different response to combined stresses; cancelled: transcript response to either or both individual stresses individual stresses returned to control levels; prioritized: opposing responses to the individual stresses and one stress response prioritized in stress combination; independent: response to only one single stress and a similar response to combined stresses; similar: similar response to combined stresses; similar: similar responses to both individual stresses and to combined stresses.
(PDF)

S3 Fig Correlation between selection on plastic responses and on expression levels in the treatment. The individuals used in this study were members of full-sib families that were split across conditions (see Material and Methods). We used the differences in family means in control and treatment condition as estimate for plasticity. To infer selection acting on plasticity when individuals live in treatment conditions, we correlated this estimate with the mean family fitness in treatment conditions. P-values are based on 10,000 permutations. A: Dry; B: Hot; C: Hot-Dry.
(PDF)

S4 Fig Coefficients of variation (CV) for all genes in each condition. Read counts were normalized to counts per million using TMM normalization. Significance of differences in the median CV between Control and stress treatments conditions were determined by permutations (10,000). CV for all genes in both conditions were randomly assigned to Control or treatment and the difference in the median was calculated. The P-value gives the proportion of permutations where the differences in median were higher than the observed difference. Control-Dry: P = 0.9252; Control-Hot: P<0.001; Control–Hot-Dry: P = 0.0028.
(PDF)

S1 Table Reproduction related genes. (XLSX)

S2 Table Changes in expression levels of genes involved in reproduction. (XLSX)

S3 Table Number of reads per sample and average quality. (XLSX)

## References

[1] Prud ‘homme B, Gompel N, Carroll SB. Emerging principles of regulatory evolution. Proc Natl Acad Sci. 2007;104: 8605–8612. 10.1073/pnas.0700488104 17494759 PMC1876436

[2] Romero IG, Ruvinsky I, Gilad Y. Comparative studies of gene expression and the evolution of gene regulation. Nat Rev Genet. 2012;13: 505–516. 10.1038/nrg3229 22705669 PMC4034676

[3] Fay JC, Wittkopp PJ. Evaluating the role of natural selection in the evolution of gene regulation. Heredity. 2008;100: 191–199. 10.1038/sj.hdy.6801000 17519966

[4] Carroll SB. Evo-Devo and an Expanding Evolutionary Synthesis: A Genetic Theory of Morphological Evolution. Cell. 2008;134: 25–36. 10.1016/j.cell.2008.06.030 18614008

[5] Long Y, Li L, Li Q, He X, Cui Z. Transcriptomic characterization of temperature stress responses in larval zebrafish. PLoS One. 2012;7 10.1371/journal.pone.0037209 22666345 PMC3364249

[6] Cui X, Affourtit J, Shockley KR, Woo Y, Churchill GA. Inheritance patterns of transcript levels in F1 hybrid mice. Genetics. 2006;174: 627–637. 10.1534/genetics.106.060251 16888332 PMC1602077

[7] Ayroles JF, Carbone MA, Stone EA, Jordan KW, Lyman RF, Magwire MM, et al Systems genetics of complex traits in *Drosophila melanogaster*. Nat Genet. 2009;41: 299–307. 10.1038/ng.332 19234471 PMC2752214

[8] Skelly DA, Ronald J, Akey JM. Inherited Variation in Gene Expression. Annu Rev Genomics Hum Genet. 2009;10: 313–332. 10.1146/annurev-genom-082908-150121 19630563

[9] Morris MRJ, Richard R, Leder EH, Barrett RDH, Aubin-Horth N, Rogers SM. Gene expression plasticity evolves in response to colonization of freshwater lakes in threespine stickleback. Mol Ecol. 2014;23: 3226–3240. 10.1111/mec.12820 24889067

[10] McCairns RJS, Smith S, Sasaki M, Bernatchez L, Beheregaray LB. The adaptive potential of subtropical rainbowfish in the face of climate change: Heritability and heritable plasticity for the expression of candidate genes. Evol Appl. 2016;9: 531–545. 10.1111/eva.12363 27099620 PMC4831457

[11] Feder ME, Walser JC. The biological limitations of transcriptomics in elucidating stress and stress responses. J Evol Biol. 2005;18: 901–910. 10.1111/j.1420-9101.2005.00921.x 16033562

[12] Evans TG. Considerations for the use of transcriptomics in identifying the “genes that matter” for environmental adaptation. J Exp Biol. 2015;218: 1925–1935. 10.1242/jeb.114306 26085669

[13] Giaever G, Chu AM, Ni L, Connelly C, Riles L, Véronneau S, et al Functional profiling of the *Saccharomyces cerevisiae* genome. Nature. 2002;418: 387–391. 10.1038/nature00935 12140549

[14] Keren L, Hausser J, Lotan-Pompan M, Vainberg Slutskin I, Alisar H, Kaminski S, et al Massively Parallel Interrogation of the Effects of Gene Expression Levels on Fitness. Cell. 2016;166: 1282–1294.e18. 10.1016/j.cell.2016.07.024 27545349

[15] Sato MP, Makino T, Kawata M. Natural selection in a population of *Drosophila melanogaster* explained by changes in gene expression caused by sequence variation in core promoter regions. BMC Evol Biol. 2016;16: 1–12. 10.1186/s12862-015-0575-y 26860869 PMC4748610

[16] Townsend JP, Cavalieri D, Hartl DL. Population genetic variation in genome-wide gene expression. Mol Biol Evol. 2003;20: 955–963. 10.1093/molbev/msg106 12716989

[17] Fraser HB. Gene expression drives local adaptation in humans Gene expression drives local adaptation in humans. Genome Res. 2013;23: 1089–1096. 10.1101/gr.152710.112 23539138 PMC3698502

[18] Hutter S, Saminadin-Peter SS, Stephan W, Parsch J. Gene expression variation in African and European populations of Drosophila melanogaster. Genome Biol. 2008;9: R12 10.1186/gb-2008-9-1-r12 18208589 PMC2395247

[19] Dayan DI, Crawford DL, Oleksiak MF. Phenotypic plasticity in gene expression contributes to divergence of locally adapted populations of *Fundulus heteroclitus*. Mol Ecol. 2015;24: 3345–3359. 10.1111/mec.13188 25847331

[20] Ghalambor CK, Hoke KL, Ruell EW, Fischer EK, Reznick DN, Hughes KA. Non-adaptive plasticity potentiates rapid adaptive evolution of gene expression in nature. Nature. 2015;525: 372–375. 10.1038/nature15256 26331546

[21] McCairns RJSS, Bernatchez L. Adaptive divergence between freshwater and marine sticklebacks: insights into the role of phenotypic plasticity from an integrated analysis of candidate gene expression. Evolution. 2009;64: 1029–1047. 10.1111/j.1558-5646.2009.00886.x 19895556

[22] Zheng W, Gianoulis TA, Karczewski KJ, Zhao H, Snyder M. Regulatory Variation Within and Between Species. Annu Rev Genomics Hum Genet. 2011;12: 327–346. 10.1146/annurev-genom-082908-150139 21721942

[23] Riehle MM, Bennett AF, Lenski RE, Long AD. Evolutionary changes in heat-inducible gene expression in lines of *Escherichia coli* adapted to high temperature. Physiol Genomics. 2003;14: 47–58. 10.1152/physiolgenomics.00034.2002 12672900

[24] Telonis-Scott M, Hallas R, McKechnie SW, Wee CW, Hoffmann AA. Selection for cold resistance alters gene transcript levels in *Drosophila melanogaster*. J Insect Physiol. 2009;55: 549–555. 10.1016/j.jinsphys.2009.01.010 19232407

[25] Yampolsky LY, Glazko G V., Fry JD. Evolution of gene expression and expression plasticity in long-term experimental populations of *Drosophila melanogaster* maintained under constant and variable ethanol stress. Mol Ecol. 2012;21: 4287–4299. 10.1111/j.1365-294X.2012.05697.x 22774776 PMC3654693

[26] Huang Y, Agrawal AF. Experimental Evolution of Gene Expression and Plasticity in Alternative Selective Regimes. PLoS Genet. 2016;12: 1–23. 10.1371/journal.pgen.1006336 27661078 PMC5035091

[27] Ehrenreich IM, Pfennig DW. Genetic assimilation: A review of its potential proximate causes and evolutionary consequences. Ann Bot. 2016;117: 769–779. 10.1093/aob/mcv130 26359425 PMC4845796

[28] Chevin LM, Lande R, Mace GM. Adaptation, plasticity, and extinction in a changing environment: Towards a predictive theory. PLoS Biol. 2010;8 10.1371/journal.pbio.1000357 20463950 PMC2864732

[29] Lande R. Adaptation to an extraordinary environment by evolution of phenotypic plasticity and genetic assimilation. J Evol Biol. 2009;22: 1435–1446. 10.1111/j.1420-9101.2009.01754.x 19467134

[30] Gasch AP, Spellman PT, Kao CM, Carmel-Harel O, Eisen MB, Storz G, et al Genomic expression programs in the response of yeast cells to environmental changes. Mol Biol Cell. 2000;11: 4241–4257. 10.1091/mbc.11.12.4241 11102521 PMC15070

[31] Gibson G. The environmental contribution to gene expression profiles. Nat Rev Genet. 2008;9: 575 10.1038/nrg2383 18574472

[32] López-Maury L, Marguerat S, Bähler J. Tuning gene expression to changing environments: From rapid responses to evolutionary adaptation. Nat Rev Genet. 2008;9: 583–593. 10.1038/nrg2398 18591982

[33] Des Marais DL, Hernandez KM, Juenger TE. Genotype-by-Environment Interaction and Plasticity: Exploring Genomic Responses of Plants to the Abiotic Environment. Annu Rev Ecol Evol Syst. 2013;44: 5–29. 10.1146/annurev-ecolsys-110512-135806

[34] Price TD, Qvarnström A, Irwin DE. The role of phenotypic plasticity in driving genetic evolution. Proc R Soc B Biol Sci. 2003;270: 1433–1440. 10.1098/rspb.2003.2372 12965006 PMC1691402

[35] Fitzpatrick BM. Underappreciated consequences of phenotypic plasticity for ecological speciation. Int J Ecol. 2012;2012: 32–37. 10.1155/2012/256017

[36] Yeh PJ, Price TD. Adaptive Phenotypic Plasticity and the Successful Colonization of a Novel Environment. Am Nat. 2004;164: 531–542. 10.1086/423825 15459883

[37] Pavey SA, Collin H, Nosil P, Rogers SM. The role of gene expression in ecological speciation. Ann N Y Acad Sci. 2010;1206: 110–129. 10.1111/j.1749-6632.2010.05765.x 20860685 PMC3066407

[38] Schneider RF, Meyer A. How plasticity, genetic assimilation and cryptic genetic variation may contribute to adaptive radiations. Mol Ecol. 2017;26: 330–350. 10.1111/mec.13880 27747962

[39] Sørensen JG, Dahlgaard J, Loeschcke V. Genetic variation in thermal tolerance among natural populations of *Drosophila buzzatii*: down regulation of Hsp70 expression and variation in heat stress resistance traits. Funct Ecol. 2001;15: 289–296. 10.1046/j.1365-2435.2001.00525.x

[40] Ghalambor CK, McKay JK, Carroll SP, Reznick DN. Adaptive versus non-adaptive phenotypic plasticity and the potential for contemporary adaptation in new environments. Funct Ecol. 2007;21: 394–407. 10.1111/j.1365-2435.2007.01283.x

[41] Whitehead A, Crawford DL. Neutral and adaptive variation in gene expression. Proc Natl Acad Sci United States Am. 2006;103: 5425–5430. 10.1073/pnas.0507648103 16567645 PMC1414633

[42] Leder EH, McCairns RJS, Leinonen T, Cano JM, Viitaniemi HM, Nikinmaa M, et al The evolution and adaptive potential of transcriptional variation in sticklebacks—Signatures of selection and widespread heritability. Mol Biol Evol. 2015;32: 674–689. 10.1093/molbev/msu328 25429004 PMC4327155

[43] Moya A, Ganot P, Furla P, Sabourault C. The transcriptomic response to thermal stress is immediate, transient and potentiated by ultraviolet radiation in the sea anemone *Anemonia viridis*. Mol Ecol. 2012;21: 1158–1174. 10.1111/j.1365-294X.2012.05458.x 22288383

[44] Moya A, Huisman L, Forêt S, Gattuso JP, Hayward DC, Ball EE, et al Rapid acclimation of juvenile corals to CO2-mediated acidification by upregulation of heat shock protein and Bcl-2 genes. Mol Ecol. 2015;24: 438–452. 10.1111/mec.13021 25444080

[45] Enzor LA, Place SP. Is warmer better? Decreased oxidative damage in notothenioid fish after long-term acclimation to multiple stressors. J Exp Biol. 2014;217: 3301–3310. 10.1242/jeb.108431 25013114

[46] Huth TJ, Place SP. Marine Genomics RNA-seq reveals a diminished acclimation response to the combined effects of ocean acidi fi cation and elevated seawater temperature in Pagothenia borchgrevinki. Mar Genomics. 2016;28: 87–97. 10.1016/j.margen.2016.02.004 26969095

[47] Schlichting CD, Smith H. Phenotypic plasticity: linking molecular mechanisms with evolutionary outcomes. Evol Ecol. 2002;16: 189–211. 10.1023/A:1019624425971

[48] Crain CM, Kroeker K, Halpern BS. Interactive and cumulative effects of multiple human stressors in marine systems. Ecol Lett. 2008;11: 1304–1315. 10.1111/j.1461-0248.2008.01253.x 19046359

[49] Byrne M, Przeslawski R. Multistressor impacts of warming and acidification of the ocean on marine invertebrates’ life histories. Integr Comp Biol. 2013;53: 582–596. 10.1093/icb/ict049 23697893

[50] Gunderson AR, Armstrong EJ, Stillman JH. Multiple Stressors in a Changing World: The Need for an Improved Perspective on Physiological Responses to the Dynamic Marine Environment. Ann Rev Mar Sci. 2016;8: 357–378. 10.1146/annurev-marine-122414-033953 26359817

[51] Kelly MW, DeBiasse MB, Villela VA, Roberts HL, Cecola CF. Adaptation to climate change: trade-offs among responses to multiple stressors in an intertidal crustacean. Evol Appl. 2016;9: 1147–1155. 10.1111/eva.12394 27695522 PMC5039327

[52] DeBiasse MB, Kelly MW. Plastic and evolved responses to global change: What can we learn from comparative transcriptomics? J Hered. 2016;107: 71–81. 10.1093/jhered/esv073 26519514

[53] Pörtner HO, Bennett AF, Bozinovic F, Clarke A, Lardies MA, Lucassen M, et al Trade‐Offs in Thermal Adaptation: The Need for a Molecular to Ecological Integration. Physiol Biochem Zool. 2006;79: 295–313. 10.1086/499986 16555189

[54] Crispo E. The Baldwin effect and genetic assimilation: Revisiting two mechanisms of evolutionary change mediated by phenotypic plasticity. Evolution. 2007;61: 2469–2479. 10.1111/j.1558-5646.2007.00203.x 17714500

[55] Healy TM, Schulte PM. Phenotypic plasticity and divergence in gene expression. Mol Ecol. 2015;24: 3220–3222. 10.1111/mec.13246 26096949

[56] Scoville AG, Pfrender ME. Phenotypic plasticity facilitates recurrent rapid adaptation to introduced predators. Proc Natl Acad Sci. 2010;107: 4260–4263. 10.1073/pnas.0912748107 20160080 PMC2840169

[57] Mäkinen H, Papakostas S, Vøllestad LA, Leder EH, Primmer CR. Plastic and evolutionary gene expression responses are correlated in European grayling (*Thymallus thymallus*) subpopulations adapted to different thermal environments. Journal of Heredity. 2016 pp. 82–89. 10.1093/jhered/esv069 26297731

[58] Gibbons TC, Metzger DCH, Healy TM, Schulte PM. Gene expression plasticity in response to salinity acclimation in threespine stickleback ecotypes from different salinity habitats. Mol Ecol. 2017;26: 2711–2725. 10.1111/mec.14065 28214359

[59] Garland T, Kelly SA. Phenotypic plasticity and experimental evolution. J Exp Biol. 2008;211: 2725–2725. 10.1242/jeb.022673 16731811

[60] Conover DO, Duffy TA, Hice LA. The covariance between genetic and environmental influences across ecological gradients: Reassessing the evolutionary significance of countergradient and cogradient variation. Ann N Y Acad Sci. 2009;1168: 100–129. 10.1111/j.1749-6632.2009.04575.x 19566705

[61] Grether GF. Environmental Change, Phenotypic Plasticity, and Genetic Compensation. Am Nat. 2005;166: E115–E123. 10.1086/432023 16224697

[62] Sokoloff A. The biology of Tribolium with special emphasis on genetic aspects I. Clarendon Press and Oxford Univ. Press, Oxford. 1972 10.1086/407731

[63] Park Y, Beeman RW. Postgenomics of Tribolium: Targeting the endocrine regulation of diuresis. Entomol Res. 2008;38: 93–100. 10.1111/j.1748-5967.2008.00143.x

[64] Milutinović B, Stolpe C, Peuß R, Armitage SAO, Kurtz J. The Red Flour Beetle as a Model for Bacterial Oral Infections. PLoS One. 2013;8 10.1371/journal.pone.0064638 23737991 PMC3667772

[65] Koch EL, Guillaume F. (2020) Data from: Fitness data of Tribolium castaneum in four different climate conditions. Dryad Digital Repository. 10.5061/dryad.gf1vhhmkn

[66] Park Y, Aikins J, Wang LJ, Beeman RW, Oppert B, Lord JC, et al Analysis of transcriptome data in the red flour beetle, *Tribolium castaneum*. Insect Biochem Molec Biol. 2008;38: 380–386. 10.1016/10.1016/j.ibmb.2007.09.008 18342244 PMC2387101

[67] Hauser F, Cazzamali G, Williamson M, Park Y, Li B, Tanaka Y, et al A genome-wide inventory of neurohormone GPCRs in the red flour beetle *Tribolium castaneum*. Front Neuroendocrinol. 2008;29: 142–165. 10.1016/j.yfrne.2007.10.003 18054377

[68] Li B, Predel R, Neupert S, Hauser F, Tanaka Y, Cazzamali G, et al Genomics, transcriptomics, and peptidomics of neuropeptides and protein hormones in the red flour beetle *Tribolium castaneum*. Genome Res. 2008;18: 113–122. 10.1101/gr.6714008 18025266 PMC2134770

[69] Aikins MJ, Schooley DA, Begum K, Detheux M, Beeman RW, Park Y. Vasopressin-like peptide and its receptor function in an indirect diuretic signaling pathway in the red flour beetle. Insect Biochem Mol Biol. 2008;38: 740–748. 10.1016/j.ibmb.2008.04.006 18549960

[70] Robinson MD, McCarthy DJ, Smyth GK. edgeR: a Bioconductor package for differential expression analysis of digital gene expression data. Bioinformatics. 2010;26: 139–140. 10.1093/bioinformatics/btp616 19910308 PMC2796818

[71] Rasmussen S, Barah P, Suarez-Rodriguez MC, Bressendorff S, Friis P, Costantino P, et al Transcriptome Responses to Combinations of Stresses in Arabidopsis. PLANT Physiol. 2013;161: 1783–1794. 10.1104/pp.112.210773 23447525 PMC3613455

[72] Ihmels J, Friedlander G, Bergmann S, Sarig O, Ziv Y, Barkai N. Revealing modular organization in the yeast transcriptional network. Nat Genet. 2002;31: 370–377. 10.1038/ng941 12134151

[73] Barabási AL, Oltvai ZN. Network biology: Understanding the cell’s functional organization. Nat Rev Genet. 2004;5: 101–113. 10.1038/nrg1272 14735121

[74] Langfelder P, Horvath S. WGCNA: An R package for weighted correlation network analysis. BMC Bioinformatics. 2008;9 10.1186/1471-2105-9-9 19114008 PMC2631488

[75] Schwenke RA, Lazzaro BP, Wolfner MF. Reproduction–Immunity Trade-Offs in Insects. Annu Rev Entomol. 2016;61: 239–256. 10.1146/annurev-ento-010715-023924 26667271 PMC5231921

[76] Parthasarathy R, Palli SR. Molecular analysis of nutritional and hormonal regulation of female reproduction in the red flour beetle, *Tribolium castaneum*. Insect Biochem Mol Biol. 2011;41: 294–305. 10.1016/j.ibmb.2011.01.006 21288489 PMC3066291

[77] Parthasarathy R, Sheng Z, Sun Z, Palli SR. Ecdysteroid regulation of ovarian growth and oocyte maturation in the red flour beetle, *Tribolium castaneum*. Insect Biochem Mol Biol. 2010;40: 429–439. 10.1016/j.ibmb.2010.04.002 20385235 PMC2916939

[78] Parthasarathy R, Sun Z, Bai H, Palli SR. Juvenile hormone regulation of vitellogenin synthesis in the red flour beetle, *Tribolium castaneum*. Insect Biochem Mol Biol. 2010;40: 405–414. 10.1016/j.ibmb.2010.03.006 20381616 PMC2875371

[79] Xu J, Tan A, Palli SR. The function of nuclear receptors in regulation of female reproduction and embryogenesis in the red flour beetle, *Tribolium castaneum*. J Insect Physiol. 2010;56: 1471–1480. 10.1016/j.jinsphys.2010.04.004 20416316 PMC2918696

[80] Lande R, Arnold SJ. The Measurement of Selection on Correlated Characters. Evolution. 1983;37: 1210 10.1111/j.1558-5646.1983.tb00236.x 28556011

[81] Falconer DS, MacKay TFC. Introduction to Quantitative Genetics. 4th Editio. Essex: Longman Group; 1996.

[82] Tufail M, Takeda M. Insect vitellogenin/lipophorin receptors: Molecular structures, role in oogenesis, and regulatory mechanisms. J Insect Physiol. 2009;55: 88–104. 10.1016/j.jinsphys.2009.01.009 19071131

[83] Kültz D. Molecular and Evolutionary Basis of the Cellular Stress Response. Annu Rev Physiol. 2005;67: 225–257. 10.1146/annurev.physiol.67.040403.103635 15709958

[84] Kassahn KS, Crozier RH, Pörtner HO, Caley MJ. Animal performance and stress: Responses and tolerance limits at different levels of biological organisation. Biol Rev. 2009;84: 277–292. 10.1111/j.1469-185X.2008.00073.x 19344429

[85] Flatt T, Tu M-P, Tatar M. Hormonal pleiotropy and the juvenile hormone regulation of Drosophila development and life history. BioEssays. 2005;27: 999–1010. 10.1002/bies.20290 16163709

[86] Gruntenko NE, Rauschenbach IY. The role of insulin signalling in the endocrine stress response in Drosophila melanogaster: A mini-review. Gen Comp Endocrinol. 2017;258: 134–139. 10.1016/j.ygcen.2017.05.019 28554733

[87] Feder ME, Hoffman GE. Heat-shock proteins, molecular chaperones, and the stress response: evolutionary and ecological physiology. Annu Rev Physiol. 1999;61: 243–282. 10.1146/annurev.physiol.61.1.243 10099689

[88] Silbermann R, Tatar M. Reproductive costs of heat shock protein in transgenic *Drosophila melanogaster*. Evolution. 2000;54: 2038–2045. 10.1111/j.0014-3820.2000.tb01247.x 11209780

[89] King B, Denholm B. Malpighian tubule development in the red flour beetle (Tribolium castaneum). Arthropod Struct Dev. 2014;43: 605–613. 10.1016/j.asd.2014.08.002 25242057

[90] Fischer EK, Ghalambor CK, Hoke KL. Can a Network Approach Resolve How Adaptive vs Nonadaptive Plasticity Impacts Evolutionary Trajectories? Integr Comp Biol. 2016;56: 877–888. 10.1093/icb/icw087 27400976

[91] Paaby AB, Rockman M V. Cryptic genetic variation: Evolution’s hidden substrate. Nat Rev Genet. 2014;15: 247–258. 10.1038/nrg3688 24614309 PMC4737706

[92] Folt C, Chen C. Synergism and antagonism among multiple stressors. Limnol Oceanogr. 1999;44: 864–877. 10.4319/lo.1999.44.3

[93] Neven LG. Physiological responses of insects to heat. Postharvest Biol Technol. 2000;21: 103–111. 10.1016/S0925-5214(00)00169-1

[94] Nguyen TTA, Michaud D, Cloutier C. A proteomic analysis of the aphid *Macrosiphum euphorbiae* under heat and radiation stress. Insect Biochem Mol Biol. 2009;39: 20–30. 10.1016/j.ibmb.2008.09.014 19000926

[95] Levine MT, Eckert ML, Begun DJ. Whole-genome expression plasticity across tropical and temperate *Drosophila melanogaster* populations from eastern Australia. Mol Biol Evol. 2011;28: 249–256. 10.1093/molbev/msq197 20671040 PMC3002243

[96] Chen X, Stillman JH. Multigenerational analysis of temperature and salinity variability affects on metabolic rate, generation time, and acute thermal and salinity tolerance in *Daphnia pulex*. J Therm Biol. 2012;37: 185–194. 10.1016/j.jtherbio.2011.12.010

[97] Sokolova IM. Energy-limited tolerance to stress as a conceptual framework to integrate the effects of multiple stressors. Integr Comp Biol. 2013;53: 597–608. 10.1093/icb/ict028 23615362

[98] Liess M, Foit K, Knillmann S, Schäfer RB, Liess HD. Predicting the synergy of multiple stress effects. Sci Rep. 2016;6: 1–8. 10.1038/s41598-016-0001-8 27609131 PMC5017025

[99] Morris MRJ, Rogers SM. Overcoming maladaptive plasticity through plastic compensation. Curr Zool. 2013;59: 526–536. 10.1093/czoolo/59.4.526

[100] Ho W-C, Zhang J. Evolutionary adaptations to new environments generally reverse plastic phenotypic changes. Nat Commun. 2018;9: 1–11. 10.1038/s41467-017-02088-w 29367589 PMC5783951

[101] Pigliucci M, Murren CJ, Schlichting CD. Phenotypic plasticity and evolution by genetic assimilation. J Exp Biol. 2006;209: 2362–2367. 10.1242/jeb.02070 16731812

[102] King AM, MacRae TH. Insect Heat Shock Proteins During Stress and Diapause. Annu Rev Entomol. 2015;60: 59–75. 10.1146/annurev-ento-011613-162107 25341107

[103] Walsh B, Blows MW. Abundant Genetic Variation + Strong Selection = Multivariate Genetic Constraints: A Geometric View of Adaptation. Annu Rev Ecol Evol Syst. 2009;40: 41–59. 10.1146/annurev.ecolsys.110308.120232

[104] Stinchcombe JR, Simonsen AK, Blows MW. Estimating uncertainty in multivariate responses to selection. Evolution. 2014;68: 1188–1196. 10.1111/evo.12321 24274331

[105] Robertson A. A mathematical model of the culling process in dairy cattle. Anim Sci. 1966;8: 95–108.

[106] Morrissey MB, Kruuk LEB, Wilson AJ. The danger of applying the breeder’s equation in observational studies of natural populations. J Evol Biol. 2010;23: 2277–2288. 10.1111/j.1420-9101.2010.02084.x 20831731

[107] Rausher MD. The measurement of selection on quantitative traits—biases due to environmental covariances between traits and fitness. Evolution. 1992;46: 616–626. 10.1111/j.1558-5646.1992.tb02070.x 28568666

[108] Bates D, Mächler M, Bolker B, Walker S. Fitting Linear Mixed-Effects Models Using lme4. J Stat Softw. 2015;67: 1–48. 10.18637/jss.v067.i01

[109] R Core Team. R: A Language and Environment for Statistical Computing. R Foundation for Statistical Computing 2015 10.1007/978-3-540-74686-7

[110] Dobin A, Davis CA, Schlesinger F, Drenkow J, Zaleski C, Jha S, et al STAR: ultrafast universal RNA-seq aligner. Bioinformatics. 2013;29: 15–21. 10.1093/bioinformatics/bts635 23104886 PMC3530905

[111] Liao Y, Smyth GK, Shi W. FeatureCounts: An efficient general purpose program for assigning sequence reads to genomic features. Bioinformatics. 2014;30: 923–930. 10.1093/bioinformatics/btt656 24227677

[112] Hatakeyama M, Opitz L, Russo G, Qi W, Schlapbach R, Rehrauer H. SUSHI: An exquisite recipe for fully documented, reproducible and reusable NGS data analysis. BMC Bioinformatics. 2016;17: 1–9. 10.1186/s12859-015-0844-1 27255077 PMC4890512

[113] Benjamini Y, Hochberg Y. Controlling the False Discovery Rate: A Practical and Powerful Approach to Multiple Testing Testing. J R Stat Soc Ser B. 1995;57: 289–300.

[114] Love MI, Anders S, Huber W. Differential analysis of count data—the DESeq2 package. Genome Biology. 2014 10.1186/s13059-014-0550-8

[115] Wu D, Lim E, Vaillant F, Asselin-Labat M-L, Visvader JE, Smyth GK. ROAST: rotation gene set tests for complex microarray experiments. Bioinformatics. 2010;26: 2176–82. 10.1093/bioinformatics/btq401 20610611 PMC2922896

[116] Reimand J, Arak T, Vilo J. g:Profiler—A web server for functional interpretation of gene lists (2011 update). Nucleic Acids Res. 2011;39: W307–W315. 10.1093/nar/gkr378 21646343 PMC3125778

[117] Szklarczyk D, Franceschini A, Wyder S, Forslund K, Heller D, Huerta-Cepas J, et al STRING v10: Protein-protein interaction networks, integrated over the tree of life. Nucleic Acids Res. 2015;43: D447–D452. 10.1093/nar/gku1003 25352553 PMC4383874

[118] Brodie ED III, Moore AJ, Janzen FJ. Visualizing and quantifying natural selection. Trends Ecol Evol. 1995;10: 313–318. </References> 10.1016/s0169-5347(00)89117-x 21237054

