# Supplementary material for: Additive and mostly adaptive plastic responses of gene expression to multiple stress in Tribolium castaneum
Source: PLoS Genet. 2020 May 7;16(5):e1008768. doi: 10.1371/journal.pgen.1008768 (PMC7238888; doi:10.1371/journal.pgen.1008768)
Supplement: S1 Appendix — (PDF) [file pgen.1008768.s001.pdf]

S1 Appendix: Comparison edgeR and Deseq2

A

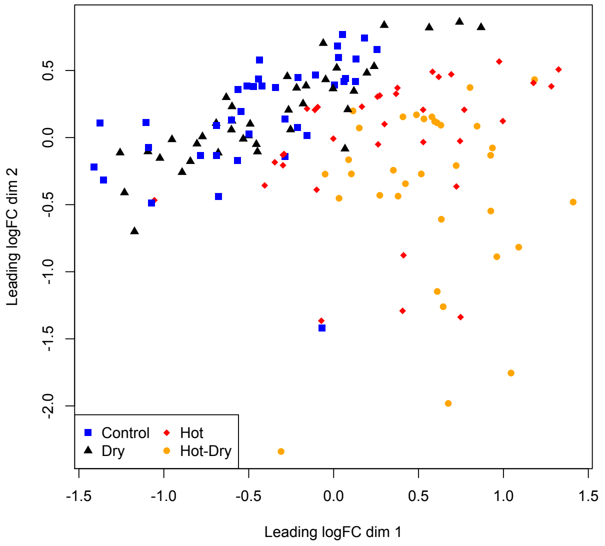

B

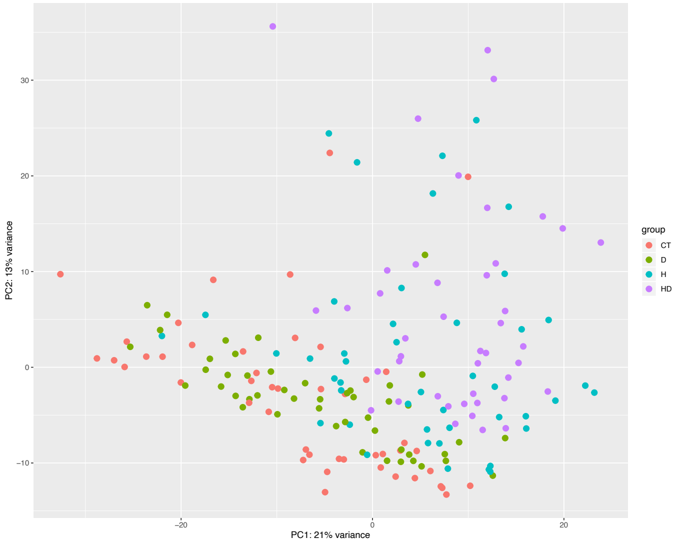

Figure S2.1: PCA plots of gene expression produced by edgeR (Robinson et al. 2010) **(A)** and by DeSeq2 (Love et al. 2014) **(B).** Each dot represents one sample. Gene expression was measured in four different climate conditions: Control (CT) 33°C, 70% relative humidity; Dry (D): 33°C, 30% r.h.; Hot (H): 37°C, 70% r.h.; Hot-Dry (HD): 37°C, 30% r.h.

Table S2.1: Number of differently expressed genes (FDR < 5%) when comparing different conditions. Up: higher expression in second condition. Down: lower expression in second condition. Differential expression analysis was conducted in DeSeq2 (Love et al. 2014). Results for the same analysis in edgeR can be found in Table 2 in the main text.

	Up	Down	total
Dry vs Control	59	66	125
Hot vs Control	1687	1355	3042
Hot.Dry vs Control	1956	1796	3752
Hot vs Dry	2153	1724	3877
Hot-Dry vs Dry	2114	1809	3923
Hot-Dry vs Hot	127	203	330

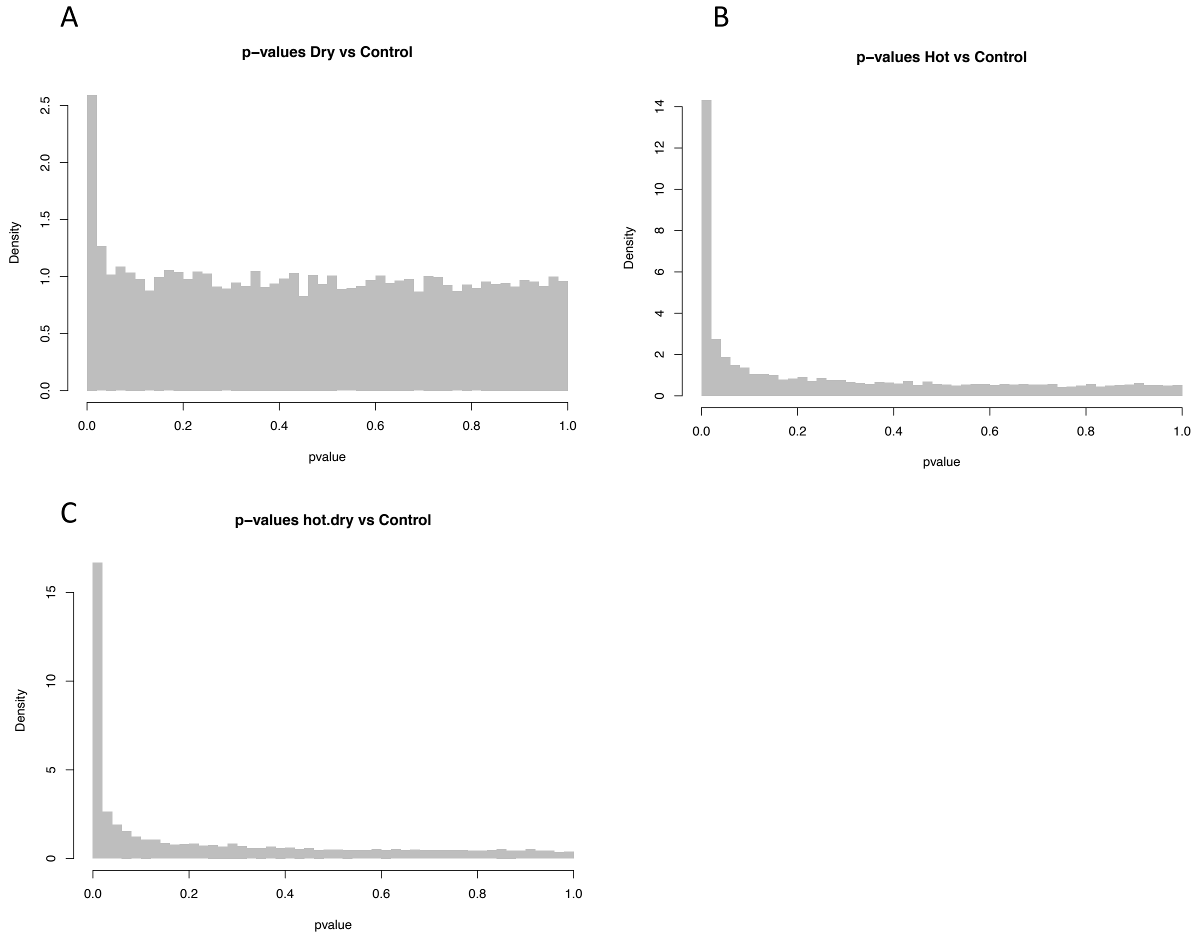

Figure S2.2: Distribution of p-values for differential gene expression under different climate conditions in three different contrasts: Dry versus Control **(A);** Hot versus Control **(B)**; Hot-Dry versus Control **(C).** The peak close to zero indicates a true signal in our data, thus confirming that our treatments conditions had an influence on gene expression levels.

References

Love, Michael, Simon Anders, and Wolfgang Huber. "Differential analysis of count data–the DESeq2 package." *Genome Biol* 15.550 (2014): 10-1186.

Robinson, Mark D., Davis J. McCarthy, and Gordon K. Smyth. "edgeR: a Bioconductor package for differential expression analysis of digital gene expression data." *Bioinformatics* 26.1 (2010): 139-140.
