# Supplementary material for: Additive and mostly adaptive plastic responses of gene expression to multiple stress in Tribolium castaneum
Source: PLoS Genet. 2020 May 7;16(5):e1008768. doi: 10.1371/journal.pgen.1008768 (PMC7238888; doi:10.1371/journal.pgen.1008768)
Supplement: S4 Appendix — (PDF) [file pgen.1008768.s004.pdf]

Coexpression analysis

Methods

Instead of focusing on single genes, a gene co-expression analysis can provide more insight into molecular mechanisms underlying trait variation. Genes do not act in isolation but are organized in pathways or functional networks with complex interactions (Ihmels et al. 2002, Barabasi and Oltvai 2004). Furthermore, considering modules instead of genes help to avoid the problem of multiple testing, since it reduces the high-dimensional data set to a few modules that are further tested for relationships with phenotypic traits

One objective we had was to test, whether certain pathways or biological processes were perturbed in stressful conditions. Additionally, we were interested to identify modules that were most relevant for fitness in the different conditions and to see whether these modules and their relationship with fitness was preserved when environmental conditions were changing.

Prior to analysis, read counts were normalized to counts per million (cpm) using the Rpackage edgeR (Robinson et al. 2010), and filtered to exclude lowly expressed genes, keeping genes that have values above 0.5 cpm in at least 5 samples. Weighted gene co-expression analysis (WGCNA) (Zhang and Horvath 2005) implemented in the Rpackage WGCNA was used to analyze transcription profiles in the four conditions separately. Briefly, WGCNA constructs networks using the absolute value of the Pearson's correlation coefficient as the gene co-expression measure, which is raised to a power β to create the adjacency matrix. The topological overlap distance calculated from the adjacency matrix is then clustered with the average linkage hierarchical clustering. For choosing power β we used the scale-free topology criterion as proposed by Zhang and Horvath 2005, resulting in β=9 in Control, β=14 in Dry, β=14 in Hot and β=12 in Hot-Dry. We used the *blockwiseModule* function in the Rpackage WGCNA to construct a signed network using a minimum module size of 20 and a *mergeCutHeight* of 0.1 (threshold for merging modules with highly correlated module eigengenes). Other parameters remained at their default. To test whether a module was related to offspring number, the correlation between a module eigengene (corresponds to the first principal component) and offspring number was used. Module preservation statistics were computed using the *modulePreservation* function (1000 permutations) implemented in WGCNA (Langfelder et al. 2011). Network module preservation statistics quantify how density and connectivity patterns of modules defined in a reference data set are preserved in a test data set without the need to define modules in the test data set. We subsequently used modules identified in Control, Dry, Hot and Hot-Dry as references and tested their preservation in the other conditions. Module quality in the reference (how closely interconnected the nodes of a module are or how well a module is separated from other modules in the network) was also assessed using *Zsummary.qual*. This measure compares density and connectivity of a module to a random module of thousand genes. The composite measure *Zsummary* and *medianRank* were used to evaluate module preservation in other conditions. *Zsummary* is a composite statistic that combines preservation of density as well as connectivity of a module. Since *Zsummary* strongly depends on module size, it is only appropriate to examine preservation of the same module in different conditions. To compare different modules within the same condition

medianRank is more informative which gives relative preservation of modules (Langfelder et al. 2011).

Results

After excluding lowly expressed genes, 12113 genes remained for analysis. We identified nine modules in control, 17 in Dry, 25 in Hot and 21 in Hot-Dry. Of all the genes in the network, 10640 (86 %) could be assigned to a module in Control, 10471 (86 %) in Dry, 10743 (89 %) in Hot and 10690 (88 %) in Hot-Dry. Quality statistics indicated that almost all modules were of high quality (Zsummary.qual>10) only few modules had a moderate quality (Zsummary.qual between eight and ten). Modules that showed a relationship with offspring number were identified in all conditions. An enrichment analysis of genes in these modules showed that almost all were related to some functional categories (GO terms), biological pathways or protein families, indicating that they are indeed biologically meaningful. Most significant and largest significant GO annotations (for biological process or molecular function) are reported for each module in Table 1 and given in parentheses after each module's colour. Although the same genes were used in all conditions, different modules were identified suggesting that some processes are specific to certain environmental conditions and not only expression but also interaction pattern between genes changed. Testing preservation of modules in other conditions can help to identify those that are only weakly preserved and might represent some specific stress response. Thereby, it can allow us to detect differences in responses to different stressors as well as to give more insight which processes are affected.

Control modules

Eight modules were found in Control (Table 1). Four of them showed a strong negative correlation with offspring number: The yellow (most significant enrichment: extracellular region) with hub gene TC005383 (ankyrin repeat and fibronectin type-III domain-containing protein 1), brown (regulation of RNA metabolic process) with TC002741, a lyosomal alpha-glucosidase, as hub gene and the blue module (protein binding). Hub gene TC008833 of that module was nucleolar GTP-binding protein 1, which is required for ribosome biogenesis (Jensen et al. 2003). Additionally, the green module (chitin metabolic process) showed a strong and highly significant negative correlation with offspring number. TC004593, a dual oxidase, was the hub gene. These enzymes can play an important role in pathogen defense (Donko et al. 2005). In contrast, there was no module with a significant positive association with fitness. Most of the modules exhibited strong preservation in the other conditions. An exception is the black module (oxidation-reduction process), which is not preserved in Hot and only weakly in Hot-Dry. Interestingly, there were also differences between conditions how well a module was preserved. The green module showed a stronger preservation in Dry and the pink module (hydrolase activity, acting on glycosyl bonds), which had the highest *medianRank* quality was well preserved in Dry and Hot, but only weakly in Hot-Dry.

Dry modules

17 modules could be detected in Dry, all of them of high quality (*Zsummary.qual* >10). Three of them showed a relationship with fitness: The green (purine ribonucleoside triphosphate binding) and brown (regulation of biological process) modules were positively correlated with offspring number, the midnightblue module (oxidation-reduction process) negatively. Interestingly, the gene that showed the second highest module membership with the green module, TC005857, Argonaute-1 *(part of the RNA silencing complex*) was assigned to the brown module indicating that both modules are regulated in a coordinated way. Most modules showed a moderate to strong preservation in the other conditions. Interestingly, the midnightblue module was only weakly preserved in Hot and Hot-Dry, but strongly in Control, whereas the salmon module (enriched in Carboxylesterase family) showed the opposite pattern.

Hot modules

25 modules were identified in Hot. Most of the modules showed moderate to strong preservation in the other conditions. Exceptions are the lightcyan (no enrichment) and lightgreen (single-organism metabolic process) module, which were not preserved in Control and Dry and only weakly in Hot-Dry. The darkred (no GO enrichment, PF08246 Cathepsin propeptide inhibitor domain (I29)) and royalblue were not preserved in any other condition. These Hot specific modules were rather small (41-74 genes) and seem to be associated with immune response. Royalblue was enriched in endopeptidase activity, specifically serine-type peptidase activity. Serine proteases are involved in immune response (Zou et al. 2006). Hub gene of the darkred module was TC000517, a pathogenesis related protein.
Level of preservation of the remaining modules differed between conditions. For example, the grey60 module showed a stronger preservation in Dry and Hot-Dry compared to Control. Lightyellow was only weakly preserved in Hot-Dry but moderately in Control and Dry, midnightblue was stronger preserved in Dry than in Control and Hot-Dry. Overall, there was not a clear pattern that Hot modules were better preserved in one certain condition.
Only the darkgrey module showed a correlation with offspring number. It showed a strong enrichment of odorant binding proteins and was also preserved in the other conditions. Interestingly, we found the same module (module with a high overlap and same enrichement) when we analysed the expression data in Dry.

Hot-Dry

Most of the modules identified in Hot-Dry showed high or moderate preservation in the other conditions. Exceptions are the royalblue (acid phosphatase activity) and darkred (hydrolase activity) modules. Similar to the Hot-specific modules they might play a role in pathogen defense. The darkred module contained bacteria binding and pathogenesis related proteins. Furthermore, it consisted of several alpha-mannosidases that can be involved in fungal digestion (Moreira et al. 2015).The royalblue module is particularly interesting since it is small and only includes 26 genes, shows a strong negative correlation with offspring number and is not preserved in Control and Dry and only weakly in Hot, whereas module quality in Hot-Dry is very high (*medianRank*=2).

DE genes and coexpression modules

Combining the two approaches of DE analysis and coexpression network construction can help us to get more insight into the processes affected by a certain stress and how interaction between genes may change. In contrast to DE analysis, which applies a stringent significance threshold and detects single genes, this enables us to examine the whole functional context to which DE genes belong. We therefore tested for an overlap between DE genes of Dry, Hot and Hot-Dry and the genes assigned to different control modules. Genes that were not DE in stress treatments were always enriched in the brown (regulation of RNA metabolic process) and turquoise module (translation). This is in agreement with our results from the module preservation statistics in which these modules showed the highest preservation. Furthermore, also in Dry, Hot and Hot-Dry modules enriched for processes related to translation were found and also the best-preserved modules. Interestingly, overlap patterns of DE genes of Hot and Hot-Dry with control modules were extremely similar: Up regulated genes were overrepresented in the green (chitin metabolic process), pink (hydrolase activity, acting on glycosyl bonds), red (hydrolase activity) and yellow (Rhodopsin-like receptor) module and among the non-assigned genes (grey module). Down-regulated genes were enriched in the blue (protein binding) module. In contrast, DE genes of Dry showed an opposite pattern: the green and red module overlapped with genes that were down regulated in Dry.

To fully understand how our different stress treatments affected functional processes, we examined relationship between control and stress modules in more detail. Additional to preservation statistics it is useful to see to which new modules genes from control modules were assigned in stress conditions and how control modules became rewired.
Most modules detected in stress conditions resulted from a further division of large control modules, resulting in a higher module number. In many cases, most of the genes in one stress module arose mainly from one control module, indicating that connectivity within that subset of a control module is preserved. For example, in Hot-Dry the turquoise control module, which was enriched in many different functional processes, became separated into the blue, greenyellow, midnightblue, tan and turquoise modules. Interestingly, greenyellow and midnightblue showed an enrichment of down-regulated genes whereas the other modules consisted mostly of genes not DE, indicating that stress affected some parts of the previously large module and thereby disconnecting them. Similar pattern can be seen in the brown and the blue module control modules. Both were enriched in a high number of different functional processes, like regulation, metabolism, signaling, gene expression. Further examination revealed that parts of these control modules involved in metabolic processes stayed closely connected and correspond to the brown and salmon module in Hot-Dry. Other parts, associated with signaling, were found in the red and black module that were both enriched in up-regulated genes. There are also examples of control modules that lost their connection in the stress treatments. Most genes of the black module (oxidation reduction process) were unassigned in Hot, most genes of the red module (hydrolase activity) were unassigned in Hot-Dry. In contrast, genes that were unassigned in control conditions formed the grey60 (odorant binding) module in Dry and darkgrey (odorant

binding) module in Hot, indicating that stress induced some correlated response. Interestingly, genes in that module were not DE relative to control condition.

Joint analysis

We conducted a joint analysis with samples from all conditions. We detected five modules and tested genes in each of them for functional enrichments using gprofiler [1]. The most significant enrichments are shown in Table 1. Subsequently, we tested the association between each module's eigengene and climate conditions (one-way ANOVA) to infer to which extent the different modules were influenced by treatment conditions. We found that the three largest modules did not show a significant association with condition (Table 1). They probably represent groups of genes involved in homeostasis and maintenance of essential cellular functions that are independent of the climate condition the individuals experience. In contrast, the two smaller modules showed a significant relationship with treatments. Next we tested for an association between each module's eigengene and fitness and how this was influenced by climate condition (interaction between eigengene and condition in a two-way ANOVA with fitness as response variable). We found that those modules that were significantly influenced by treatments showed a strong association with fitness. We further found that the effect of a module' eigengene was condition dependent.

Table A: Modules detected in joint analysis. Shown are the number of genes in each module, the p-value for testing each module's eigengene association with treatment conditions (one-way ANOVA results), the p-value for testing each module's eigengene association with fitness dependent on condition (two-way ANOVA with interaction eigengene x condition) and the most significant enriched GO biological processes and KEGG pathways.

Module	N genes	p-value treatment effect	p-value association fitness	p-value association with fitness is condition dependent	enrichment	
**turquoise**	5255	0.504	0.077	0.510	KEGG:03010	Ribosome
					KEGG:03060	Protein export
					KEGG:03040	Spliceosome
					GO:0006412	translation
					GO:0043043	peptide biosynthetic process
					GO:0022613	ribonucleoprotein complex biogenesis
					GO:0006518	peptide metabolic process
					GO:0006396	RNA processing
					GO:0042254	ribosome biogenesis
**yellow**	620	1.292E-09***	0.010**	1.36E-04***	GO:0007186	G protein-coupled receptor signaling pathway
**green**	151	1.14E-12***	2.00E-04***	0.6392	GO:0006030	chitin metabolic process
					GO:1901071	glucosamine-containing compound metabolic process
					GO:0006040	amino sugar metabolic process
**blue**	2447	0.083	0.772	6.22E-05***	GO:0051252	regulation of RNA metabolic process
					GO:0019219	regulation of nucleobase-containing compound metabolic process
					GO:0051171	regulation of nitrogen compound metabolic process
					KEGG:04330	Notch signaling pathway
					KEGG:03050	Proteasome
**brown**	1431	0.449	0.152	0.002**	no significant enrichment	

Conclusions

In Control some very large modules were detected that were enriched in a high number of different GO categories like metabolic processes, signaling, regulation, indicating that all these processes work in a coordinated way. In stress conditions these large modules became separated into smaller ones according to the different functional processes that were previously linked in non-stressful conditions.

Combining results of DE and network analysis by overlaying DE and functional modules revealed that large parts of control modules that are related to metabolism were conserved. Their genes did not show a significant change in expression and were involved in processes that are required for homeostasis. Other parts of the modules stayed closely connected, but many genes including their hub genes showed a response to stress thereby partly rewiring functional networks.

Stress treatments seem to disrupt the normally tight connections between metabolism and replication.

The effect of the different modules on fitness is condition dependent. Gene expression and its underlying network structure is thus experiencing different selection pressures in the different conditions.

References

Barabasi, A. L., & Oltvai, Z. N. (2004). Network biology: understanding the cell's functional organization. *Nature reviews genetics*, *5*(2), 101-113.

Donkó, Á., Péterfi, Z., Sum, A., Leto, T., & Geiszt, M. (2005). Dual oxidases. *Philosophical Transactions of the Royal Society B: Biological Sciences*, *360*(1464), 2301-2308.

Ihmels, J., Friedlander, G., Bergmann, S., Sarig, O., Ziv, Y., & Barkai, N. (2002). Revealing modular organization in the yeast transcriptional network. *Nature genetics*, *31*(4), 370-377.

Jensen, B. C., Wang, Q., Kifer, C. T., & Parsons, M. (2003). The NOG1 GTP-binding protein is required for biogenesis of the 60 S ribosomal subunit. *Journal of Biological Chemistry*, *278*(34), 32204-32211.

Langfelder, P., & Horvath, S. (2008). WGCNA: an R package for weighted correlation network analysis. *BMC bioinformatics*, *9*(1), 559.

Langfelder, P., Luo, R., Oldham, M. C., & Horvath, S. (2011). Is my network module preserved and reproduible?. *PLoS computational biology*, *7*(1), e1001057.

Moreira, N. R., Cardoso, C., Ribeiro, A. F., Ferreira, C., & Terra, W. R. (2015). Insect midgut α-mannosidases from family 38 and 47 with emphasis on those of Tenebrio molitor. *Insect biochemistry and molecular biology*, *67*, 94-104.

Robinson, M. D., McCarthy, D. J., & Smyth, G. K. (2010). edgeR: a Bioconductor package for differential expression analysis of digital gene expression data. *Bioinformatics*, *26*(1), 139-140.

Zhang, B., & Horvath, S. (2005). A general framework for weighted gene co-expression network analysis. *Statistical applications in genetics and molecular biology*, *4*(1).

Zou, Z., Lopez, D. L., Kanost, M. R., Evans, J. D., & Jiang, H. (2006). Comparative analysis of serine protease-related genes in the honey bee genome: possible involvement in embryonic development and innate immunity. *Insect molecular biology*, *15*(5), 603-614.

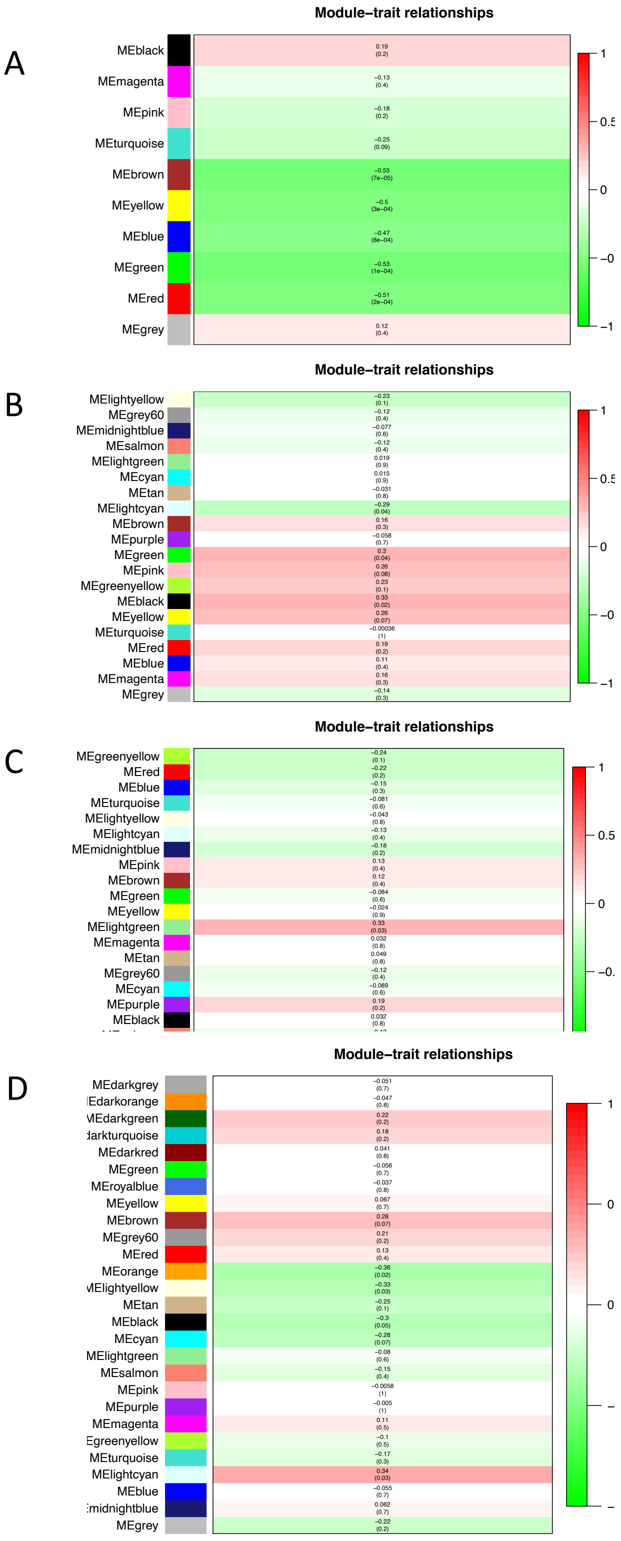

Figure A: Modules detected by WGCNA in four different conditions based on gene expression data of female *Tribolium castaneum*: A: Control; B: Dry; C: Hot; D: Hot-

Dry and their correlation with offspring number. Heat map colours refer to correlation coefficients and p-values are given in brackets.

Table B: Enrichments and preservation of functional modules detected by WGCNA in four different conditions based on gene expression data of female *Tribolium castaneum*:

		Genes	Zsummary. qual	medianRank quality	top Term	Preservation Zsummary			Preservation medianRank		
	**Control modules**					**in Dry**	**in Hot**	**in Hot-Dry**			
1	black	86	19	2	oxidation-reduction process	6.1	0.26	2.3	5	9	6
2	blue	2240	35	8	protein binding	24	20	19	7	7	6
3	brown	1665	46	6	regulation of RNA metabolic process	50	46	36	1	1	1
4	green	356	40	3	chitin metabolic process	5.1	9.9	6.8	9	6	7
5	pink	39	17	1	hydrolase activity, acting on glycosyl l	6.4	6.5	3.3	2	2	4
6	red	256	19	7	hydrolase activity	11	10	8.5	6	5	8
7	turquoise	5410	67	4	translation	33	32	33	3	3	2
8	yellow	408	28	5	extracellular region/Rhodopsin-like rec	16	17	14	4	4	4
	**Dry modules**					**in Control**	**in Hot**	**in Hot-Dry**			
1	black	133	29	3	no GO enrichment, Interpro:Zinc finger,	10	14	16	7	3	3
2	blue	2363	37	16.5	organonitrogen compound biosyntheti	33	29	27	5	8	10
3	brown	1858	36	16.5	regulation of biological process	20	25	22	11	13	15
4	cyan	55	11	11	no GO enrichment, KEGG:Lysosome	3.8	2.9	5.1	14	17	13
5	green	883	60	5.5	purine ribonucleoside triphosphate l	29	22	21	6	11	12
6	greenyellow	94	13	13	serine-type endopeptidase activity	4.2	5.9	7	16	12	11
7	grey60	32	14	2	odorant binding	6.3	8.1	7	3	2	1
8	lightcyan	46	13	6	monooxygenase activity	5.4	7.7	8.7	7	6	3
9	magenta	127	17	12	no GO enrichment, KEGG:Starch and suc	5.4	8.2	9.4	16	13	8
10	midnightblue	54	13	9	oxidation-reduction process	6.3	2.6	2.9	6	17	14
11	pink	129	33	1	intracellular transport	16	16	15	1	2	3
12	purple	109	21	7	no enrichment	4.4	6.8	6.7	13	11	12
13	red	221	20	15	serine-type peptidase activity	9.5	11	8.7	10	11	16
14	salmon	78	18	4.5	no GO enrichment, Pfam: Carboxylestera	4.2	11	9.5	14	4	6
15	tan	85	17	8	oxidation-reduction process	3.5	6.7	3	17	13	17
16	turquoise	2387	58	10	peptide metabolic process	35	45	41	2	4	5
17	yellow	1817	48	14	regulation of RNA metabolic process	24	35	30	9	7	9
	**Hot modules**					**in Control**	**in Dry**	**in Hot-Dry**			
1	black	322	37	16	no enrichment	11	8.7	13	16	21	15
2	blue	2350	54	12	regulation of nitrogen compound met	24	38	29	12	6	11
3	brown	1921	40	6	protein binding	31	28	30	6	13	13
4	cyan	91	16	20	monooxygenase activity	3.7	6	5.3	20	15	18
5	darkgreen	40	15	15	no enrichment	2.7	6	5.7	15	12	10
6	darkgrey	36	14	6	odorant binding	5.4	8	6.9	6	3	4
7	darkred	41	19	26	no GO enrichment, PF08246 Cathepsin p	0.36	0.28	2	26	25	26
8	darkturquoise	39	11	11	no enrichment	4.2	4.9	8	11	13	3
9	green	610	37	10	organonitrogen compound biosynthe	18	18	23	10	17	11
10	greenyellow	109	26	4	ATP binding	8.3	11	7.7	4	6	7
11	grey60	69	27	20	organonitrogen compound metabolic	2.7	9.8	8.4	20	5	8
12	lightcyan	74	19	25	no enrichment	-0.011	-0.16	3.6	25	26	21
13	lightgreen	63	17	23	single-organism metabolic process	1.1	1	2.6	23	24	20
14	lightyellow	55	14	8	hydrolase activity	4.3	7.3	2	8	9	23
15	magenta	268	31	7	protein binding	14	17	13	7	5	12
16	midnightblue	76	21	9	no enrichment	7.4	13	8.1	9	2	9
17	orange	30	13	11	chitin binding	3.4	2.9	2.5	11	20	21
18	pink	272	36	10	plasma membrane part	12	11	14	10	14	6
19	purple	153	18	14	chitin binding	8	9.9	9.5	14	15	11
20	red	553	44	5	intracellular	21	21	20	5	10	10
21	royalblue	41	10	23	endopeptidase activity	0.63	1.7	1.1	23	23	25
22	salmon	100	18	15	catalytic activity	5.2	9.6	7	15	11	18
23	tan	104	18	19	no enrichment	4.9	6	5.2	19	18	19
24	turquoise	2698	65	3	intracellular ribonucleoprotein comp	34	42	43	3	4	2
25	yellow	628	28	17	no enrichment	16	26	22	17	10	14
						**in Control**	**in Dry**	**in Hot**			
	**Hot-Dry modules**										
1	black	355	38	10	single organism signaling	11	7.2	12	16	20	19
2	blue	1654	64	10	organonitrogen compound biosynthe	35	41	41	5	4	5
3	brown	1443	50	16	regulation of RNA metabolic process	25	42	41	11	5	3
4	cyan	134	22	10.5	no enrichment	8.1	11	12	13	11	9
5	darkred	21	12	3	hydrolase activity	2.2	2	2.7	10	14	19
6	green	613	37	14	binding	20	26	20	6	6	13
7	greenyellow	212	38	4.5	protein binding	16	16	18	2	5	4
8	grey60	60	16	7.5	monooxygenase activity	4.8	7.9	7.1	11	8	8
9	lightcyan	100	22	8	DNA replication, CC: MCM complex	9.7	16	11	7	1	6
10	lightgreen	59	9.9	18	organic acid metabolic process	3.1	4	4.9	19	16	16
11	lightyellow	54	8.3	21	no GO enrichment, protein domain: CRA	3	3.7	7	18	15	10
12	magenta	339	48	4.5	no enrichment	8.7	8.2	10	17	19	19
13	midnightblue	119	26	6	DNA replication	14	16	13	2	3	4
14	pink	354	23	20	oxidoreductase activity	6.3	5.6	8.4	20	21	21
15	purple	311	32	12	protein binding	14	13	14	8	15	14
16	red	581	42	13	voltage-gated potassium channel a	14	18	19	15	12	14
17	royalblue	26	15	2	acid phosphatase activity	0.16	0.23	2.1	22	20	15
18	salmon	151	18	15	nitrogen compound metabolic process	8.3	9.2	9.9	12	12	13
19	tan	157	42	1	amide biosynthetic process	13	12	16	4	10	2
20	turquoise	3155	48	17	chromosome segregation	29	27	30	6	12	9
21	yellow	792	35	19.5	binding	20	33	26	13	8	11
