# Supplementary material for: Additive and mostly adaptive plastic responses of gene expression to multiple stress in Tribolium castaneum
Source: PLoS Genet. 2020 May 7;16(5):e1008768. doi: 10.1371/journal.pgen.1008768 (PMC7238888; doi:10.1371/journal.pgen.1008768)
Supplement: S1 Fig — Control: 33°C, 70% relative humidity, N = 1575; Dry: 33°C, 30% r.h., N = 1642; Hot: 37°C, 70% r.h., N = 1401; Hot-Dry: 37°C, 30% r.h., N = 1567. (PDF) [file pgen.1008768.s006.pdf]

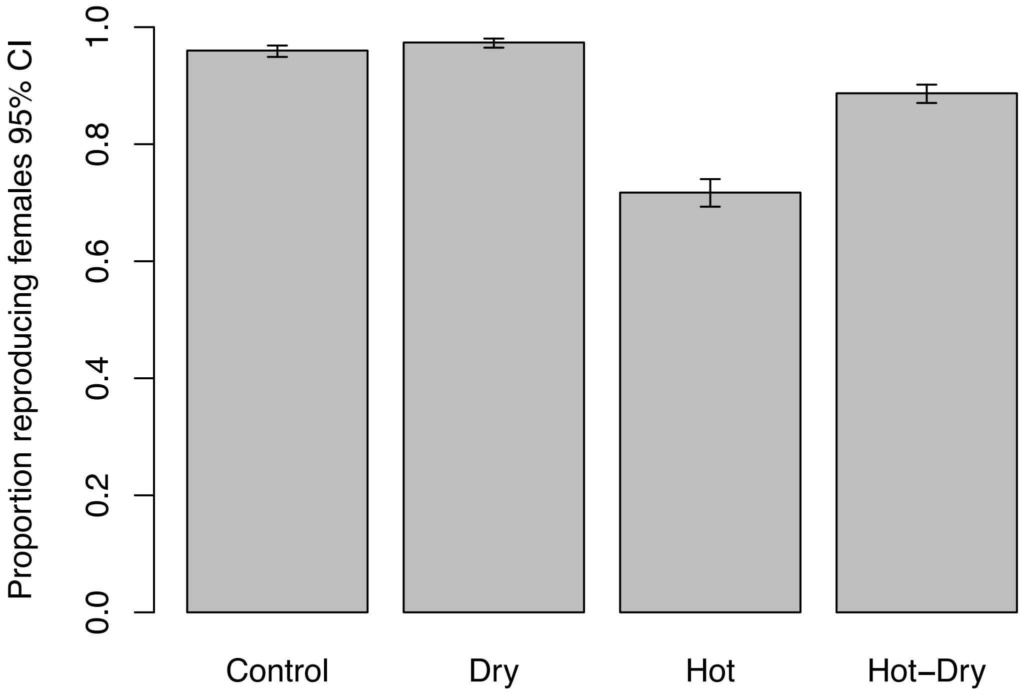

S1 Figure: Proportion of reproducing females in four different conditions. **Control**: 33°C, 70% relative humidity, N=1575; **Dry**: 33°C, 30 % r.h., N=1642; **Hot**: 37°C, 70 % r.h., N=1401; **Hot-Dry**: 37°C, 30 % r.h., N= 1567
