# Supplementary material for: Additive and mostly adaptive plastic responses of gene expression to multiple stress in Tribolium castaneum
Source: PLoS Genet. 2020 May 7;16(5):e1008768. doi: 10.1371/journal.pgen.1008768 (PMC7238888; doi:10.1371/journal.pgen.1008768)
Supplement: S2 Fig — Combinatorial: Similar levels in the two in the two individual stresses but a different response to combined stresses; cancelled: transcript response to either or both individual stresses individual stresses returned to control levels; prioritized: opposing responses to the individual stresses and one stress response prioritized in stress combination; independent: response to only one single stress and a similar response to combined stresses; similar: similar response to combined stresses; similar: similar responses to both individual stresses and to combined stresses. (PDF) [file pgen.1008768.s007.pdf]

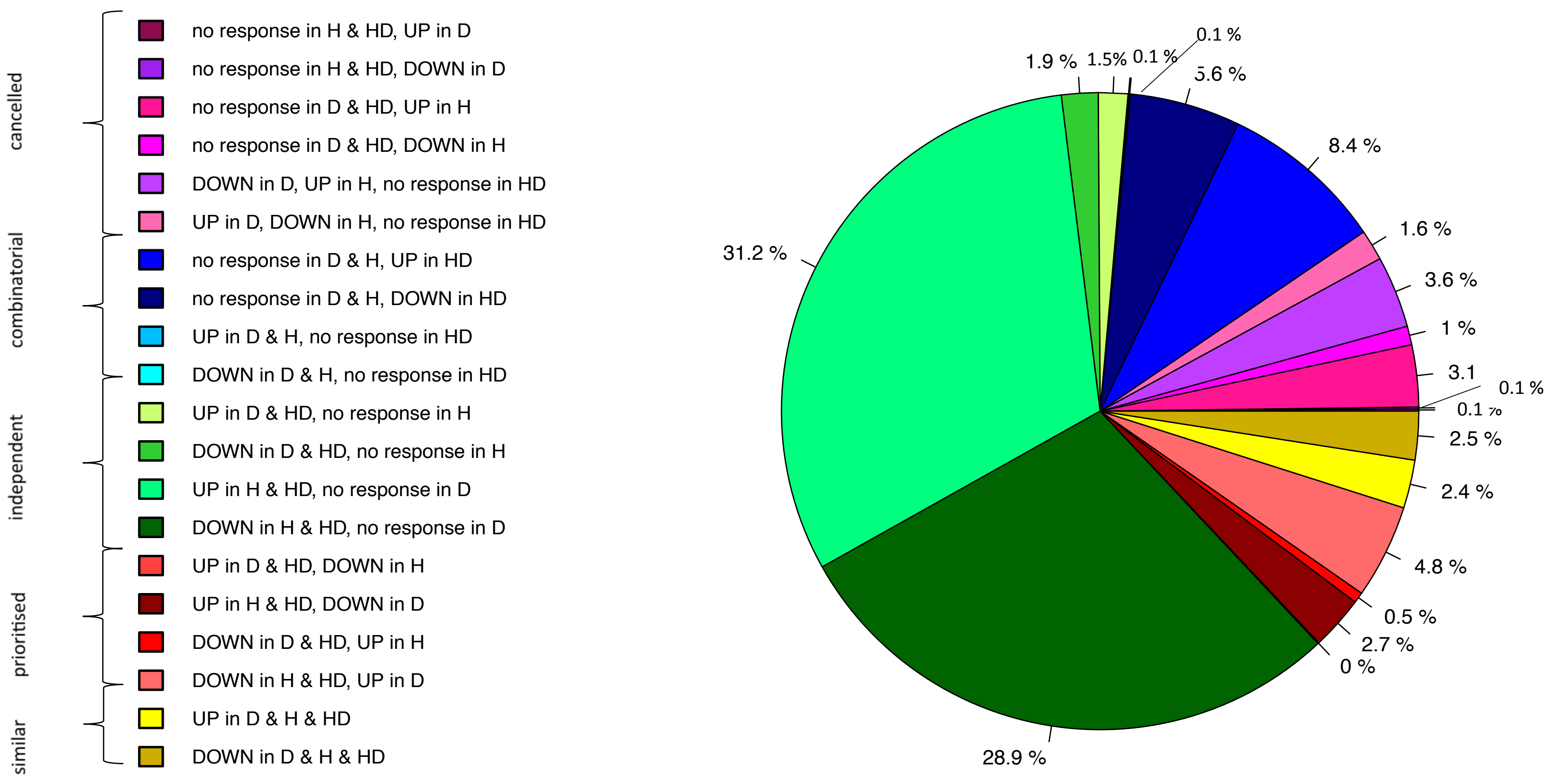

S2 Fig: Subcategories of different response modes giving more details about the most prevalent pa7erns : Response modes of significantly responding transcripts in the stress treatments (Dry (D), Hot (H), Hot-Dry (HD). Combinatorial: Similar levels in the two individual stresses but a different response to combined stresses; cancelled: transcript response to either or both individual stresses returned to control levels; prioriFzed: opposing responses to the individual stresses and one stress response prioriFzed in response to combined stresses; independent, response to only one single stress and a similar response to combined stresses; similar: similar responses to both individual stresses and to combined stresses.
