# Supplementary material for: Additive and mostly adaptive plastic responses of gene expression to multiple stress in Tribolium castaneum
Source: PLoS Genet. 2020 May 7;16(5):e1008768. doi: 10.1371/journal.pgen.1008768 (PMC7238888; doi:10.1371/journal.pgen.1008768)
Supplement: S3 Fig — The individuals used in this study were members of full-sib families that were split across conditions (see Material and Methods). We used the differences in family means in control and treatment condition as estimate for plasticity. To infer selection acting on plasticity when individuals live in treatment conditions, we correlated this estimate with the mean family fitness in treatment conditions. P-values are based on 10,000 permutations. A: Dry; B: Hot; C: Hot-Dry. (PDF) [file pgen.1008768.s008.pdf]

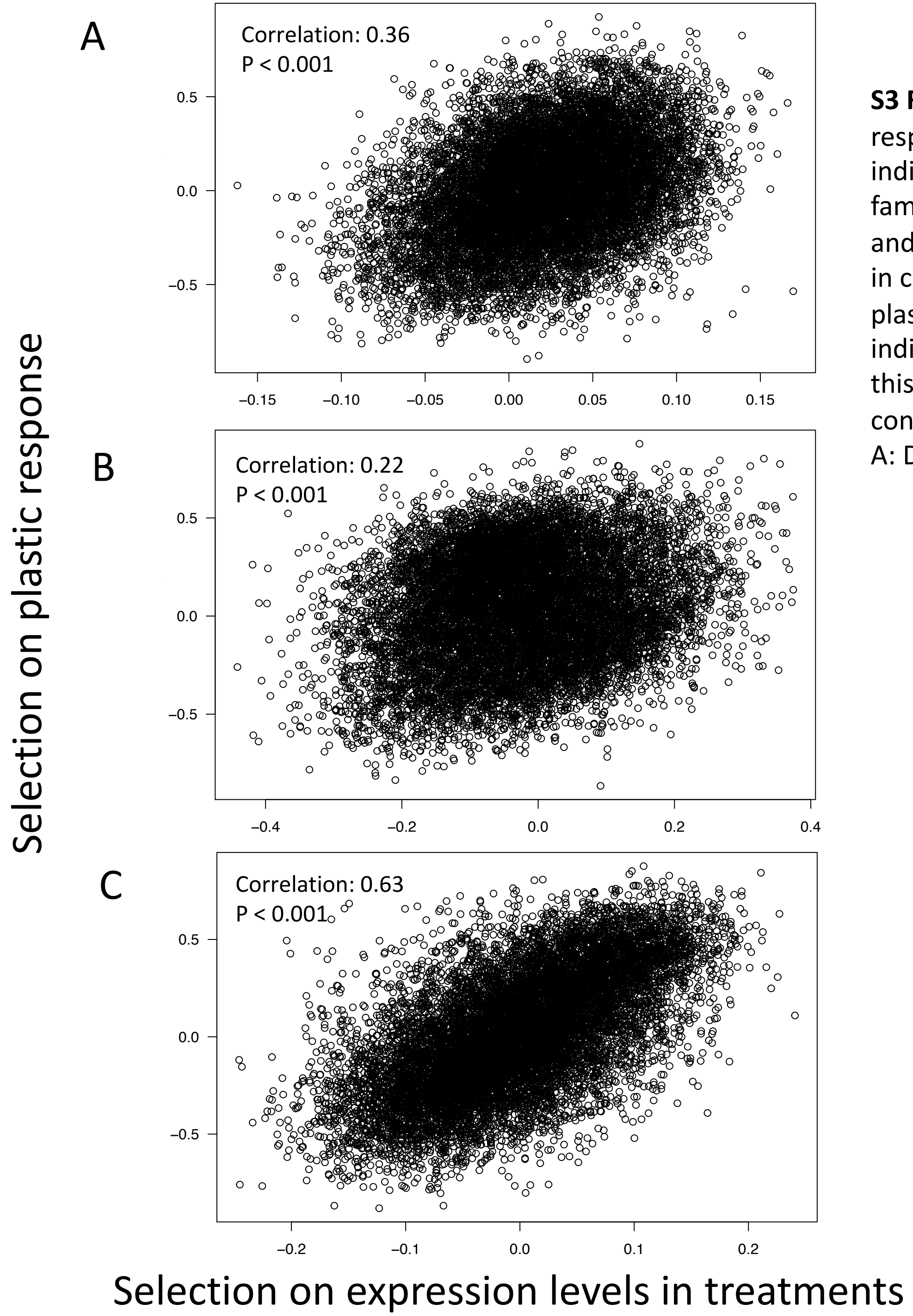

S3 Fig: Correlation between selection on plastic responses and on expression levels in the treatment. The individuals used in this study were members of full-sib families that were split across conditions (see Material and Methods). We used the differences in family means in control and treatment condition as estimate for plasticity. To infer selection acting on plasticity when individuals live in treatment conditions, we correlated this estimate with the mean family fitness in treatment conditions. P-values are based on 10,000 permutations. A: Dry; B: Hot; C: Hot-Dry
