# Supplementary material for: Additive and mostly adaptive plastic responses of gene expression to multiple stress in Tribolium castaneum
Source: PLoS Genet. 2020 May 7;16(5):e1008768. doi: 10.1371/journal.pgen.1008768 (PMC7238888; doi:10.1371/journal.pgen.1008768)
Supplement: S4 Fig — Read counts were normalized to counts per million using TMM normalization. Significance of differences in the median CV between Control and stress treatments conditions were determined by permutations (10,000). CV for all genes in both conditions were randomly assigned to Control or treatment and the difference in the median was calculated. The P-value gives the proportion of permutations where the differences in median were higher than the observed difference. Control-Dry: P = 0.9252; Control-Hot: P<0.001; Control–Hot-Dry: P = 0.0028. (PDF) [file pgen.1008768.s009.pdf]

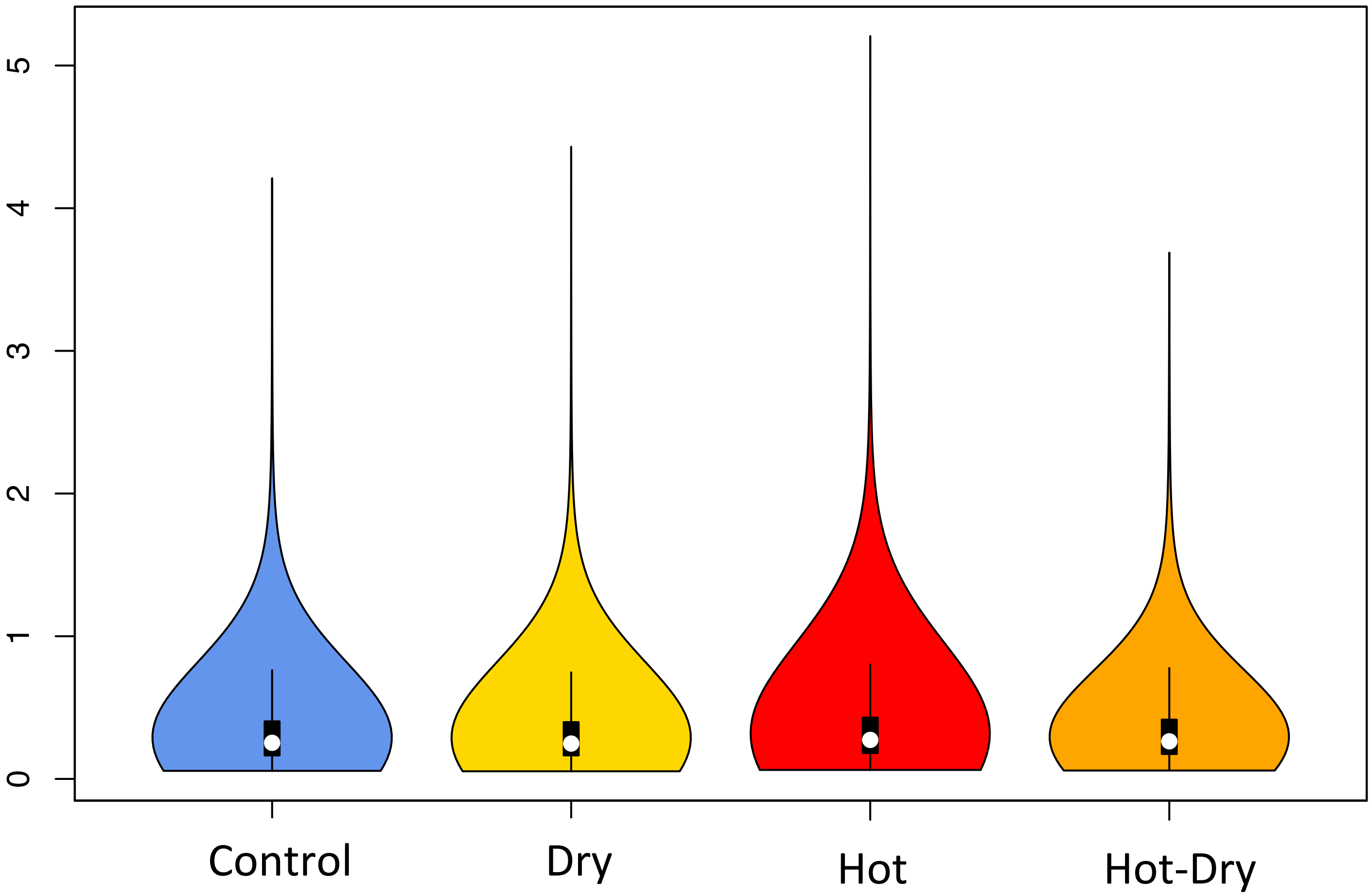

Figure S4: Coefficients of variation(CV) for all genes in each condition. Read counts were normalized to counts per million using TMM normalization. Significance of differences in the median CV between Control and stress treatments conditions were determined by permutations (10,000). CV for all genes in both conditions were randomly assigned to Control or treatment and the difference in the median was calculated. The pvalue gives the proporFon of permutations where the differences in median were higher than the observed difference. Control-Dry: P=0.9252; Control-Hot: P<0.001; Control–Hot-Dry: P=0.0028.
